# Excitable neuronal assemblies with adaptation as a building block of brain circuits for velocity-controlled signal propagation

**DOI:** 10.1371/journal.pcbi.1006216

**Published:** 2018-07-06

**Authors:** Hesam Setareh, Moritz Deger, Wulfram Gerstner

**Affiliations:** 1 School of Computer and Communication Sciences and Brain Mind Institute, School of Life Sciences, Ecole Polytechnique Fédérale de Lausanne, Switzerland; 2 Institute for Zoology, Faculty of Mathematics and Natural Sciences, University of Cologne, Köln, Germany; University of Edinburgh, UNITED KINGDOM

## Abstract

The time scale of neuronal network dynamics is determined by synaptic interactions and neuronal signal integration, both of which occur on the time scale of milliseconds. Yet many behaviors like the generation of movements or vocalizations of sounds occur on the much slower time scale of seconds. Here we ask the question of how neuronal networks of the brain can support reliable behavior on this time scale. We argue that excitable neuronal assemblies with spike-frequency adaptation may serve as building blocks that can flexibly adjust the speed of execution of neural circuit function. We show in simulations that a chain of neuronal assemblies can propagate signals reliably, similar to the well-known synfire chain, but with the crucial difference that the propagation speed is slower and tunable to the behaviorally relevant range. Moreover we study a grid of excitable neuronal assemblies as a simplified model of the somatosensory barrel cortex of the mouse and demonstrate that various patterns of experimentally observed spatial activity propagation can be explained.

## Author summary

Models of activity propagation in cortical networks have often been based on feedforward structures. Here we propose a model of activity propagation, called excitation chain, which does not need such a feedforward structure. The model is composed of excitable neural assemblies with spike-frequency adaptation, connected bidirectionally in a row or a grid. This prototypical neural circuit can propagate activity forwards, backwards or in both directions. Furthermore, the propagation speed is slow enough to trigger the generation of behaviors on the time scale of hundreds of milliseconds. A two-dimensional variant of the model is able to generate different activity propagation patterns, similar to spontaneous activity and stimulus-evoked responses in anesthetized mouse barrel cortex. We propose the excitation chain model as a basic component that can be employed in various ways to create spiking neural circuit models that generate signals on behavioral time scales. In contrast to abstract models of excitable media, our model makes an explicit link to the time scale of neuronal spikes.

## Introduction

Reliable propagation of activity is necessary for processing and transmitting sensory signals in the brain. During the last two decades, two prominent types of computational models have been studied to address this issue. First, the synfire chain consists of groups of spiking neurons connected in a feedforward architecture [1–4] potentially embedded in recurrent networks [5, 6]. Second, rate propagation models [6–9] use a similar feedforward architecture, but instead of spikes they propagate fluctuations of the firing rate. In a synfire chain the refractory behavior of neurons after firing a spike is the crucial element of stable activity propagation [10]. The derivative of the membrane potential shapes spike density and sharpens the activity pulse [10, 11]. Statistical methods [12, 13] have been proposed for detecting the firing pattern of synfire chains in spike train recordings. Reliable neuronal firing patterns compatible with synfire chains have been observed in area HVC of the song-bird [14], while the statistical significance of synfire chains in cortical neurons is questionable [15]. Synfire chain models have been used for reproducing behavioral functions such as bird song generation [16] and monkey scribbling [17]. Systems of interacting synfire chains were also used for building a large-scale model of cortex [18, 19].

While both synfire chain and rate propagation succeeded to model fast behaviors (behaviors on the order of milliseconds), they are expensive in terms of neuron numbers and therefore not suitable for reproducing behavioral phenomena that need a slower, and sometimes tunable, speed of activity propagation. Essentially synfire chains implement a clock, set by the delay of spike propagation and the rise time of excitatory postsynaptic potentials on the millisecond time scale [20, 21]. In order to address this issue, we propose an *excitation chain* model, for activity propagation in a bidirectional chain of neuronal assemblies. Our model can be considered as a spiking version of excitable media [22–26], with an explicit link to the neuronal time scale of spikes.

In contrast to previous synfire chain and rate propagation models, our model does not require an explicit feedforward architecture. Specific feedforward structures have not been observed in experiments in the neocortex so far. Here we propose an excitation chain model that is consistent with the following experimental connectivity data: first, inter- and intra-assembly connection probability and synaptic weights with values in the experimentally observed ranges [27, 28]; and second, clustered connectivity of neurons [29]. Hence, although the concept of an excitation chain as such is a rather generic and abstract model, its basic connectivity features are consistent with experimentally observed properties.

In the next section, we describe our model and its dynamics in detail. We also illustrate how activity propagation can be made faster or slower by changing synaptic weights. Then we analyze the behavior of the model and explain the role of its elements in forming the dynamics. Finally, we extend the excitation chain to a two-dimensional model, which we may call an excitation grid. We argue that this grid of assemblies can be considered as the skeleton of barrel cortex, which can generate different spatio-temporal modes of activity propagation observed experimentally in barrel cortex [30, 31].

## Results

### The speed of activity propagation in a chain of excitable bistable assemblies

**Fig 1:**
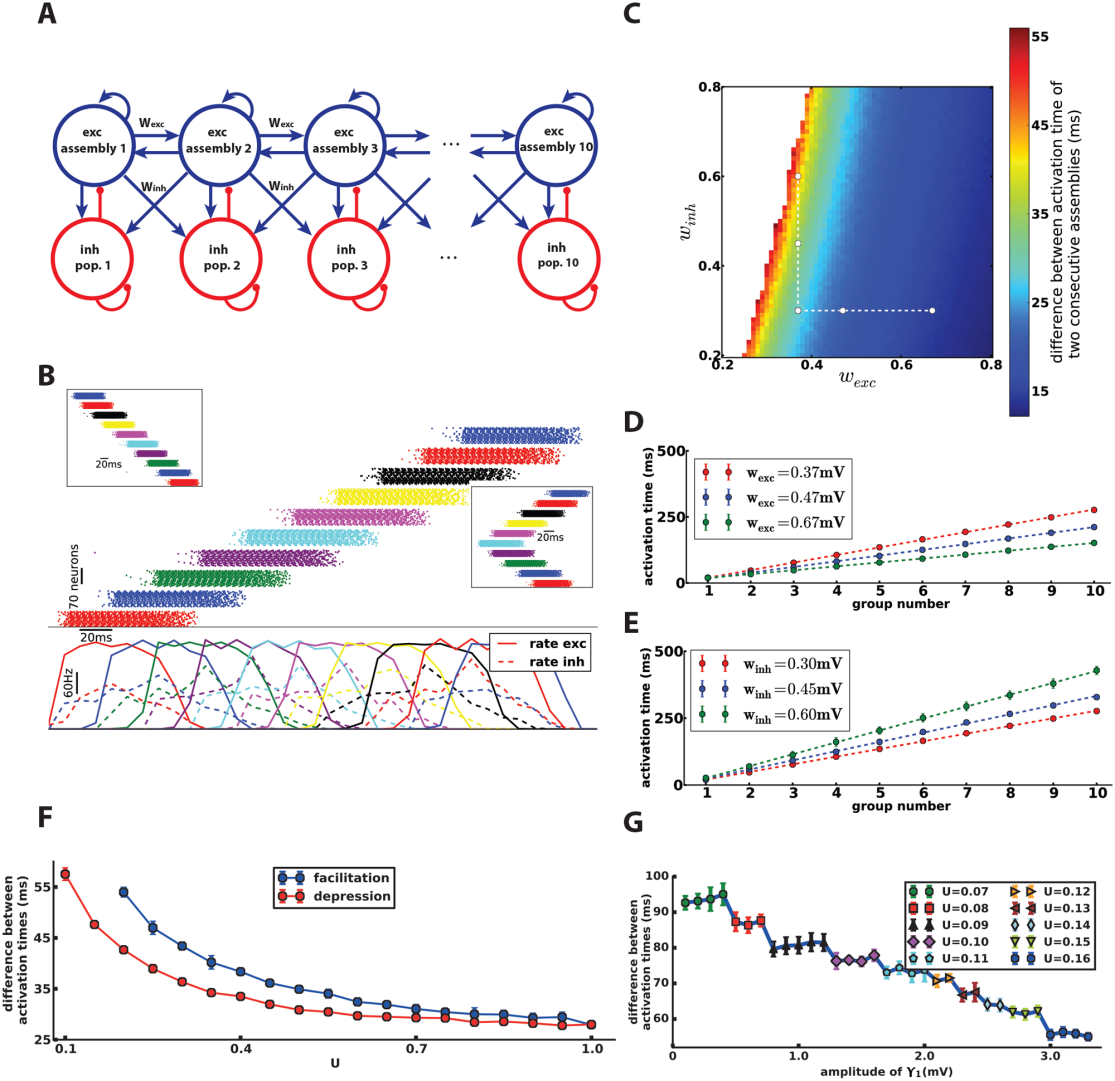
Excitation wave in a one-dimensional chain. **A**) Schematic of the excitation chain. Each excitatory assembly is connected to the excitatory assemblies and inhibitory populations of its neighboring groups in the chain, while the inhibitory population is bidirectionally connected to the excitatory assembly of its own group. **B**) spike raster of excitatory assemblies (top) and average rate of each assembly (bottom, estimated in bins of 10ms). Based on the place of the stimulation, the chain can propagate the spiking activity forwards, backwards (top-left inset) or in both directions (bottom-right inset). **C**) The difference of the activation times of two consecutive assemblies can be adjusted by changing synaptic weights. The delay is increased by increasing the weights from excitatory assemblies to their neighboring inhibitory assemblies (*w*_inh_) or by decreasing the weights between excitatory assemblies (*w*_exc_). The white area denotes the parameter region in which the chain cannot propagate the activation (The intersection of the two dashed lines in **C** denotes the parameters used in **B**). **D**)The total delay of the chain (and inversely the propagation speed) depends on *w*_exc_ (compare white dots on horizontal dashed line in **C**). **E**) The total delay also depends on *w*_inh_ (compare white dots on vertical dashed line in **C**). **F**) Regulation of propagation speed using short-term plasticity parameter. Increasing the parameter *U* decreases the difference of activation times of two consecutive assemblies for both depression and facilitation. In the case of facilitating synapses, the activity cannot propagate for low values of *U*. The other parameters of the model (including *w*_exc_ and *w*_inh_) are the same as in **B**. **G**) Decreasing the amplitude of the dynamic threshold *γ*_1_(*t*) together with a readjustment of the parameter *U* of short-term plasticity influences the difference of activation times (horizontal axis). For each value of amplitude, we found the value of parameter *U* (shown in the legend) which yields the largest delay. Every point in **C**, **D**, **E**, **F** and **G** is the mean over 10 different trials. Errorbars indicate standard deviations. The right-most circle repeats the left-most blue point in **F**.

In order to propagate a signal of activation through several excitable assemblies, we first connect several groups of neurons in a bidirectional chain (Fig 1A). Each group contains an excitatory assembly and a population of inhibitory neurons. Assemblies are defined as small populations of neurons with high connection probability. More precisely, inside each excitatory assembly synapses are strong and the connectivity is high (connection probability *p* = 50%) whereas each inhibitory population has smaller synaptic weights and lower connectivity. Within each group, the excitatory assembly and inhibitory population are mutually connected. Moreover, the excitatory assembly is connected to the inhibitory population and excitatory assembly of neighboring groups. In contrast, the inhibitory populations do not have inter-group connections (see Materials and methods for the details and parameters).

#### Basic mechanism and functionality

If we stimulate the excitatory assembly of the first group of the chain with a transient stimulus of 25ms duration (see Materials and methods), all neurons of this group fire several spikes. The activation of this first assembly is then propagated step by step through the chain of assemblies until the last group. Fig 1B shows the raster plot of all excitatory neurons as well as the population-averaged activity of excitatory and inhibitory populations of the chain. One can see that despite the reciprocal connections between excitatory assemblies, the activity is propagated in a feedforward manner. We repeated the simulation several times with different transient stimuli (S1 Fig). Whenever the transient input stimulus is able to activate the first excitatory assembly, the activity is reliably propagated through the chain to the last group. For a vast range of parameters (Fig 1C), we have observed neither a termination of the activity wave nor an instability in the propagation dynamics (such as convergence to synchronous firing of all excitatory neurons).

If we stimulate the excitatory assembly of the last group, we see that the activity propagates backwards (Fig 1.inset). The excitation wave can also spread in both direction simultaneously. Stimulating an excitatory assembly in the middle of the chain produces two traveling waves, one towards the beginning of the chain and another one towards its end (Fig 1.inset). The property of activity propagation in different directions has been observed in multi-electrode extracellular recordings of the neocortex. For example, based on the place of local application of glutamate, neural firing is initiated in a forward, backward or bidirectional manner [32].

#### Speed depends on synaptic weights

The speed of activity propagation in our excitation chain is much lower than in a synfire chain [3]. In synfire chains the time needed for the activation to jump to next group is on the order of the synaptic transmission delay (1–5ms), while in our model this time is roughly 13- 55ms, although we have used a short synaptic transmission delay of 1ms. This slow propagation allows us to describe phenomena on the time scale of several hundreds of milliseconds or even seconds.

The speed of propagation in the excitation chain is controlled by the inter-assembly synaptic weights. Let us first define how to measure the delay (and consequently the speed) of activity propagation. For the sake of simplicity, we define the activation time of each excitatory assembly by the average time of the first spike of each neuron in the assembly. Alternatively, and without change of the measured propagation delay, we could also define the activation time of an assembly of *N* neurons as the time when *N*/2 neurons have fired a first spike after an interval of at least 100ms. Importantly, the difference of the activation time of two neighboring assemblies can be considered as the time needed for transmitting the activity signal from one group to the next group. The inverse of the activation time difference of the first and the last assembly can be used as a measure for propagation speed.

We use the symbols *w*_exc_ to denote the synaptic weights between neighboring excitatory assemblies and *w*_inh_ to denote the synaptic weight from excitatory assemblies to the neighboring inhibitory populations. Fig 1C, 1D and 1E show that increasing *w*_exc_ increases the speed, while increasing *w*_inh_ reduces it. The activity wave remains stable and propagates reliably over a broad range of parameter values.

#### Removing the inhibitory populations

**Fig 2:**
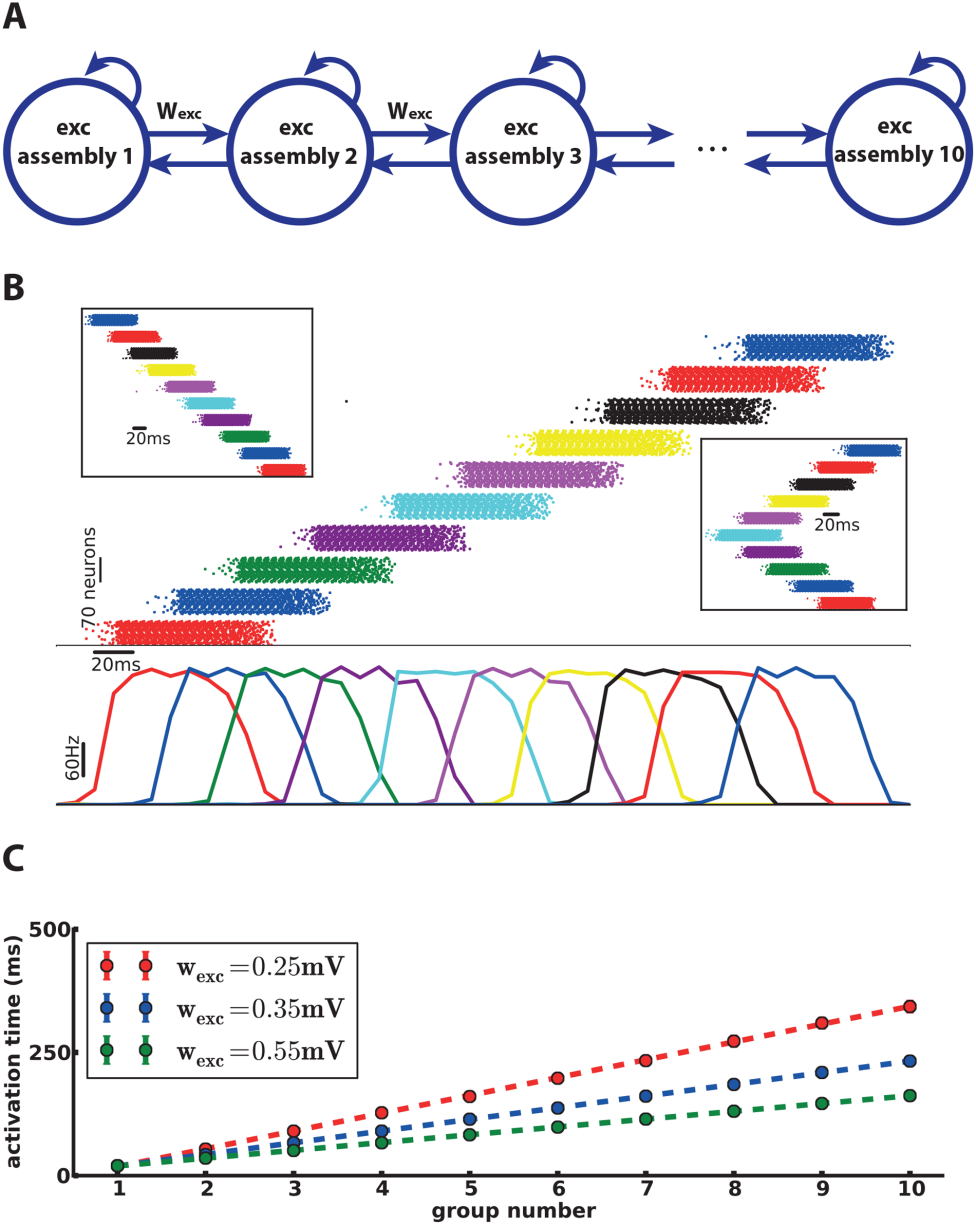
Inhibitory populations are not necessary. A chain containing only excitatory assemblies (**A**) propagates the spiking activities in both directions (**B**) similar to the case with inhibitory assemblies (Fig 1B). The propagation speed can be tuned (**C**) by modifying the synaptic weights between excitatory assemblies (*w*_exc_), albeit in a smaller range, than in Fig 1.

Excitatory assemblies are the essential elements of the excitation chain, while the role of inhibitory populations in the chain consists mostly in reducing the propagation speed. Therefore, we may simplify our model by removing all inhibitory populations. A chain of excitatory assemblies only (Fig 2A) is able to propagate the activity in different directions (Fig 2B) similar to a chain containing also inhibitory neurons. The propagation speed is regulated by modifying *w*_exc_ (Fig 2C). However, because of the lack of inhibition, the speed cannot be lower than a critical value below which transmission becomes unreliable. In our simulations, we found a maximum delay of 34ms instead of 56ms with inhibition.

### Analysis of excitation chain dynamics

#### Excitation chains rely on bistable assembly dynamics

**Fig 3:**
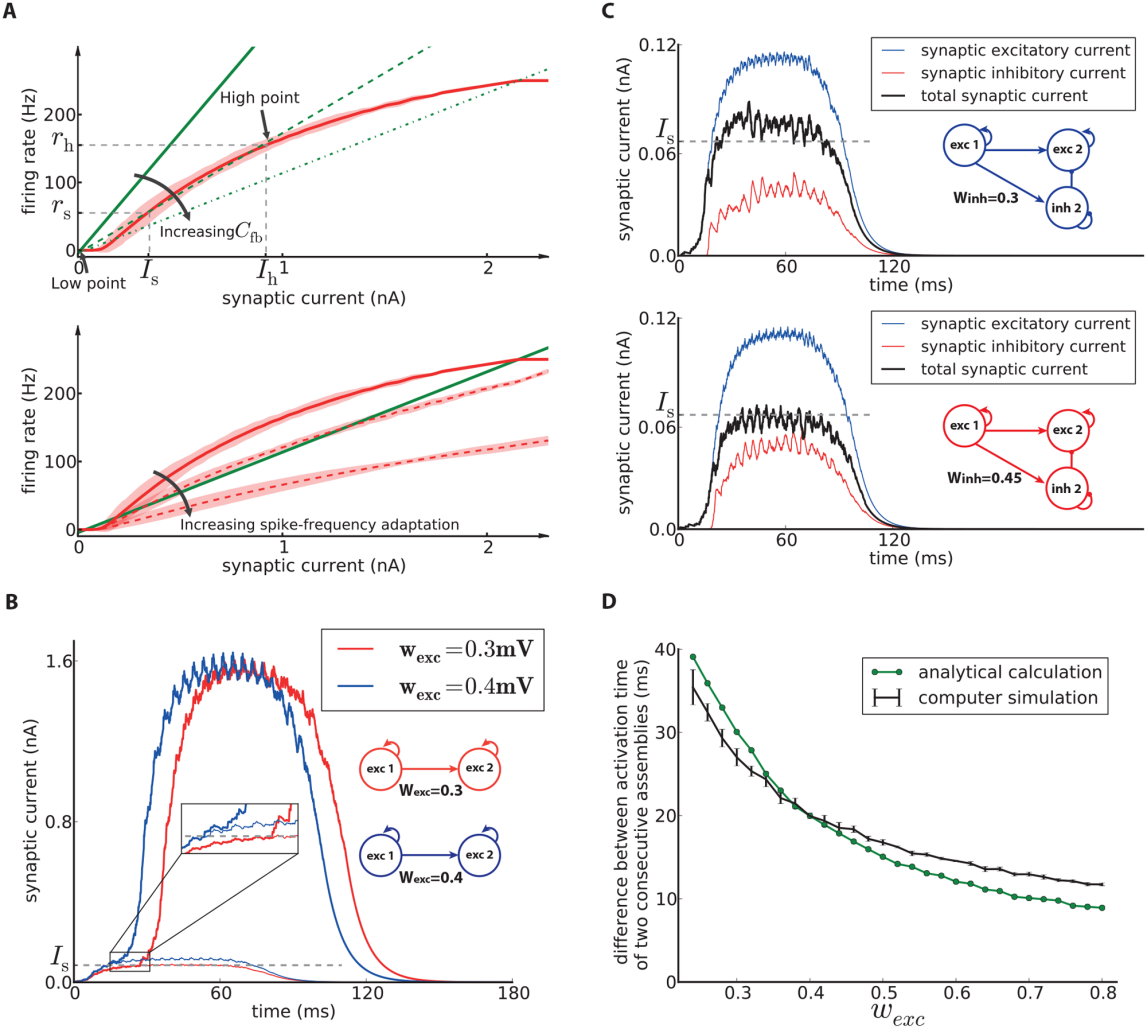
Analysis of model dynamics. **A**-Top) The network feedback (*C*_fb_, Eq 9) affects the dynamics of the system. The red curve is the noisy gain function of the GIF neuron model (mean spike count in a group of 50 independent neurons over 10ms, divided by 50 × 10ms, shaded area marks ±3 SEM) measured during the initial 10ms after switch-on of a synaptic current of mean 〈*I*_syn_〉. The green lines (solid, dashed and dash-dotted) show the relation of firing rate and synaptic current caused by network feedback (see Materials and methods, Eq 8) for increasing *C*_fb_. The slope of the green lines is inversely proportional to the effective feedback coefficient *C*_fb_ of the population. Intersections of the red curve with one of the green lines indicate potential stationary states (fixed points) of a network of non-adapting neurons. **A**-Bottom) The noisy gain function of adapting neurons during the first 10ms after stimulus onset (solid red curve) is different from that later (dashed red curves). **B**) Synaptic current received by the second assembly (averaged over the assembly’s neurons) in two chains with different inter-assembly synaptic weights. The thin lines show the averaged synaptic current each neuron receives from the previous assembly, while the thick lines show the total synaptic current received from both the assembly itself and the previous assembly. When the thick line separates from the thin line, the assembly starts to fire spikes on its own. This is the moment when the assembly crosses the switch point and approaches the high-activity fixed point. This occurs earlier if inter-assembly synaptic weights are increased (blue). Consequently the propagation speed along the excitation chain increases. **C**) The total synaptic current received by the second assembly (black) is the sum of excitation (blue) from the first assembly and inhibition (red traces show the absolute value of inhibitory currents) from the second inhibitory population. When the total input (black) crosses the threshold, the assembly switches to the high-point and becomes activated. Increasing synaptic weights from the first assembly to the second inhibitory population increases the inhibition and decreases the total input received by the second assembly. Hence it delays the activation time. **D**) The analytical approach (green line, see Materials and methods) approximates the difference between the activation time of two consecutive assemblies with an error of less than 4ms. The black line shows the value of the difference obtained by averaging over 10 simulations (error bars indicate standard deviation).

In order to understand the dynamics of the chain and identify the components that determine the propagation speed, we first focus on one assembly of excitatory neurons. The dynamics of each assembly can be described by self-consistent equations relating the firing rate of the assembly to the average synaptic input of the neurons (see Materials and methods). If the assembly has a high network feedback coefficient *C*_fb_ (Eq 9), the dynamics of the system has three fixed points (Fig 3A-top): the *low point* which is a stable fixed point with zero firing rate, the *switch point* which is the unstable middle fixed point and a high-rate fixed point which is called the *high point*. If the assembly is driven by synaptic input greater than the switch point current (*I*_s_), it approaches the high point and produces a high firing rate. Since the intra-assembly synaptic weights (*w*_exc_) and connection probability (*p*) are high, the network feedback coefficient (*C*_fb_ ∝ *pw*_exc_) is also high. Therefore, the dynamics of the assembly can be explained by this three-fixed-point configuration, which we call the *excitable mode* of the assembly. However, because of spike-frequency adaptation of our excitatory neuron model, the frequency of spike emission progressively decreases during the high-rate state. Consequently, the neurons’ gain function changes gradually (Fig 3A-bottom) and the system goes to a new configuration which has only one fixed point, the low point. (Note that, for the same reason, the assembly does not fully converge to the high point as it is shown in Fig 3A-top. While the assembly is approaching the high point, changes of the gain function move the position of the fixed point.) Therefore the assembly eventually becomes quiet and stops firing. We refer to this configuration as the *dormant mode* of the assembly. In this mode, receiving synaptic input does not activate the assembly. It takes a while for the assembly to recover from the dormant mode and return to the three-fixed-point configuration, which is the excitable mode. Previous work [33] addressed the dynamics for a simpler adaptive integrate-and-fire neuron model with similar analytical approaches.

#### Synaptic weights determine the propagation speed

For the second step of the analysis, we take into account the interaction of neighboring assemblies in the chain. Consider the case of a chain of excitatory assemblies only. When assembly 1 goes to the high point and each of its neurons fire several spikes within a short time, it sends strong synaptic input to assembly 2. However, since the synaptic weights between assemblies are relatively low, early volleys of spikes of assembly 1 do not yet suffice for assembly 2 to cross the switch point. Therefore it takes some time for assembly 2 to accumulate enough input from assembly 1. This is the reason why the propagation of activity is slow in the excitation chain and the difference of activation time is much higher than the synaptic delay. If we increase the inter-assembly weight, then a smaller number of spikes of assembly 1 is needed to produce the switch current in assembly 2, which means it will be activated sooner (Fig 3C). This explains the increase of the propagation speed along the chain when we strengthen the inter-assembly synapses (Fig 2C).

#### Inhibitory neurons delay the activation of each group

Let us now discuss the effect of adding the inhibitory populations to the chain. Each inhibitory population sends inhibitory input to the excitatory assembly in the same group and thus reduces the effect of the synaptic input provided by the neighboring excitatory assemblies. Therefore, the inhibition delays the time at which the switch current is reached. If we increase the inter-group synaptic weight onto inhibitory neurons *w*_inh_, the inhibitory neurons fire more often and produce more inhibition. This, in turn, increases the amount of synaptic input needed from the neighboring excitatory assembly and further delays the activation time. Fig 3C illustrates the effect of *w*_inh_ on the synaptic inputs.

#### Adaptation gives rises to directed activity propagation

Suppose that assembly 1 has become active and sends synaptic current to assembly 2. When excitatory assembly 2 receives enough synaptic current to cross the switch point, it becomes active at a high firing rate. It then sends synaptic current to its neighbors, excitatory assembly 1 and 3. However, by that time, excitatory assembly 1 is about to fall into the dormant mode and cannot generate many spikes. Therefore, only excitatory assembly 3 switches to the high point. This procedure repeats until the end of the chain. Therefore activity propagates in one direction only although connections between assemblies are bidirectional. An analogous situation happens for the backwards propagation. In case of stimulating an assembly in the middle of a previously quiet chain, since both of its neighbor assemblies are in the excitable mode, they both switch to the high point. Consequently, the activity propagates in both directions from then on.

Spike-frequency adaptation is responsible for the transition to the dormant mode by progressively changing the gain function (Fig 3A-bottom) during the active phase of an assembly, and eventually for its termination. By modification of the adaptation parameters of excitatory neurons, we are able to adjust the duration of the activate phase of each assembly [34].

#### The effect of other synaptic weights on the dynamics

After having analyzed the effect of inter-group synaptic weights (*w*_exc_ and *w*_inh_) on the propagation speed of the chain, we now focus on the weights of inhibitory to excitatory neurons inside the same group. Since intra-group inhibition contributes to the total inhibition of the assembly, it can have effects similar to *w*_inh_ on the propagation speed. In order to avoid redundant parameter search, we kept the intra-group inhibition constant and explored *w*_inh_. Likewise, the intra-group excitatory to excitatory connections (connections inside each assembly) are also important. As we mentioned earlier, assemblies should have a high network feedback coefficient (*C*_fb_ ∝ *pw*). Otherwise, they would not be able to switch to the high point and produce high firing rates in case of receiving relatively low synaptic input.

Both connection probability and synaptic weight of the intra-group excitatory to excitatory connections affect the network feedback coefficient and therefore shape the core of the excitation chain. Other intragroup connections (excitatory to inhibitory and inhibitory to inhibitory connections) are less important. However too much inhibition may shut down the assembly by finishing its activation rapidly so that not enough synaptic input arrives at the next assembly. Therefore, it may lead to a loss of signal propagation.

#### Obtaining the propagation speed using an analytical approach

The self-consistent approach relating the firing rate to the average synaptic input which we mentioned earlier is useful for a qualitative explanation of the dynamics of the excitation chain. However, it is not suitable for a quantitative calculation of the propagation speed, because we cannot calculate the exact gain function for this neuron model (see Materials and methods) if the effects of spike-frequency adaptation become strong. Therefore, we developed another analytical approach (see Materials and methods) in order to obtain the difference between activation times of two consecutive assemblies in the chain of only excitatory assemblies (Fig 2). Fig 3D compares the results of simulation and the analytical approach for different values of *w*_exc_. Our theory estimates the activation time difference with an error of less than 4ms.

#### Embedding short-term plasticity in the model

We can also add short-term facilitation and depression [35, 36] (see Materials and methods) to the model. In these cases, we are able to adjust the propagation speed by manipulating the parameters of facilitation and depression, while keeping *w*_exc_ and *w*_inh_ fixed. In the first variation, we added facilitation to the excitatory synapses between assemblies. The case of no facilitation that we considered earlier corresponds to a choice of *U* = 1 for the usage parameter *U* of short-term plasticity. We observed that decreasing the value of *U* to values below one increases the difference between activation times of two consecutive assemblies (Fig 1F). In the presence of facilitation, the amplitude of post-synaptic currents (PSC) is initially lower compared to case of not having facilitation. When a presynaptic neuron fires several spikes within a short interval, the amplitude of each PSC in the post-synaptic neuron increases. Only after a suitable number of spikes, the PSC amplitude reaches a stationary value. Therefore, it takes time for an assembly to provide sufficient amount of synaptic input to activate the next assembly. Modification of the recovery time constant *τ*_rec_, however, does not affect the propagation speed (S2 Fig).

In the second variation, we neglected facilitation and added depression in inter-group excitatory to inhibitory connections. We also increased the intragroup inhibitory to excitatory synaptic weight by ∼ 7 times (1.07mV instead of 0.16mV). The propagation delay decreased as we increased the value of *U* (Fig 1F). The reason is that a large amount of inhibition from the inhibitory subpopulation does not allow the excitatory assembly to become active. Depressing synapses from one assembly to the neighboring inhibitory subpopulation reduce the amount of PSC onto inhibitory subpopulation over time so that the activity of an inhibitory subpopulation and its projecting inhibition drop off. Similar to the previous case, the time constant (*τ*_facil_) does not affect the propagation speed (S2 Fig).

Note that we still need spike-frequency adaptation in the above cases because it is the adaptation that terminates the activation of the assemblies. Without adaptation, an assembly switches to active and remains active for the rest of the simulation. It is important to notice that our model is not based on a competition between assemblies via inhibition (see Discussion) and every assembly switches to the low point on its own. If we want to remove the adaptation and preserve the functionality of the chain, we can add short-term depression to intra-assembly synapses. We will come back to this point in the Discussion section.

In addition to connectivity, the duration of the active phase of assemblies plays an important role in the propagation speed. The duration should be long enough, such that each assembly can provide the synaptic input needed to activate the next assembly in the chain. Therefore, if we want to achieve a slower propagation speed, we need to increase the duration of the active phase.

The duration of the active phase can be adjusted by modification of adaptation parameters of excitatory neurons [34]. Each time a neuron fires a spike, several adaptation processes on several time scales are triggered and generate both a spike-triggered current and an increase in firing threshold [37]. To describe these adaptation effects we use two exponentially decaying kernels, *η*_*k*_(*t*) (*k* = 1, 2) for spike-triggered currents and *γ*_*k*_(*t*) (*k* = 1, 2) for dynamic threshold. In Fig 1G we decreased the amplitude of kernel *γ*_1_(*t*) (the kernel with the shorter time constant). Then we used short-term facilitation for changing the propagation speed (similar to Fig 1F). For each value of the kernel amplitude, we found the value of the usage parameter *U* which causes slowest propagation. Fig 1G summarizes the activation times of assemblies for different combinations of parameters. We achieved a maximum delay of 95ms between one assembly and the next using this approach. Hence, we conclude that a linear chain of 11 assemblies is sufficient to cover a typical behavioral time scale of above 1 second.

The dynamic threshold (*γ*(*t*)) has two decaying kernels, one with shorter time constant (tens of milliseconds) and the other one with longer time constant (several hundreds of milliseconds). The shorter time constant determines the duration of the active phase of assembly (see [34]). The longer one affects the time that an assembly needs to recover from the dormant mode after termination of active phase. The kernels of the spike-triggered current (*η*(*t*), see Materials and methods) have similar effects. In fact, we could remove one of these two mechanisms (dynamic threshold or spike-triggered current) and preserve the functionality of the chain. However, since the original model used both mechanisms [38, 39] to fit experimental data, we preferred to keep both of them.

#### Reduction of firing rate by using a second type of inhibitory neurons

**Fig 4:**
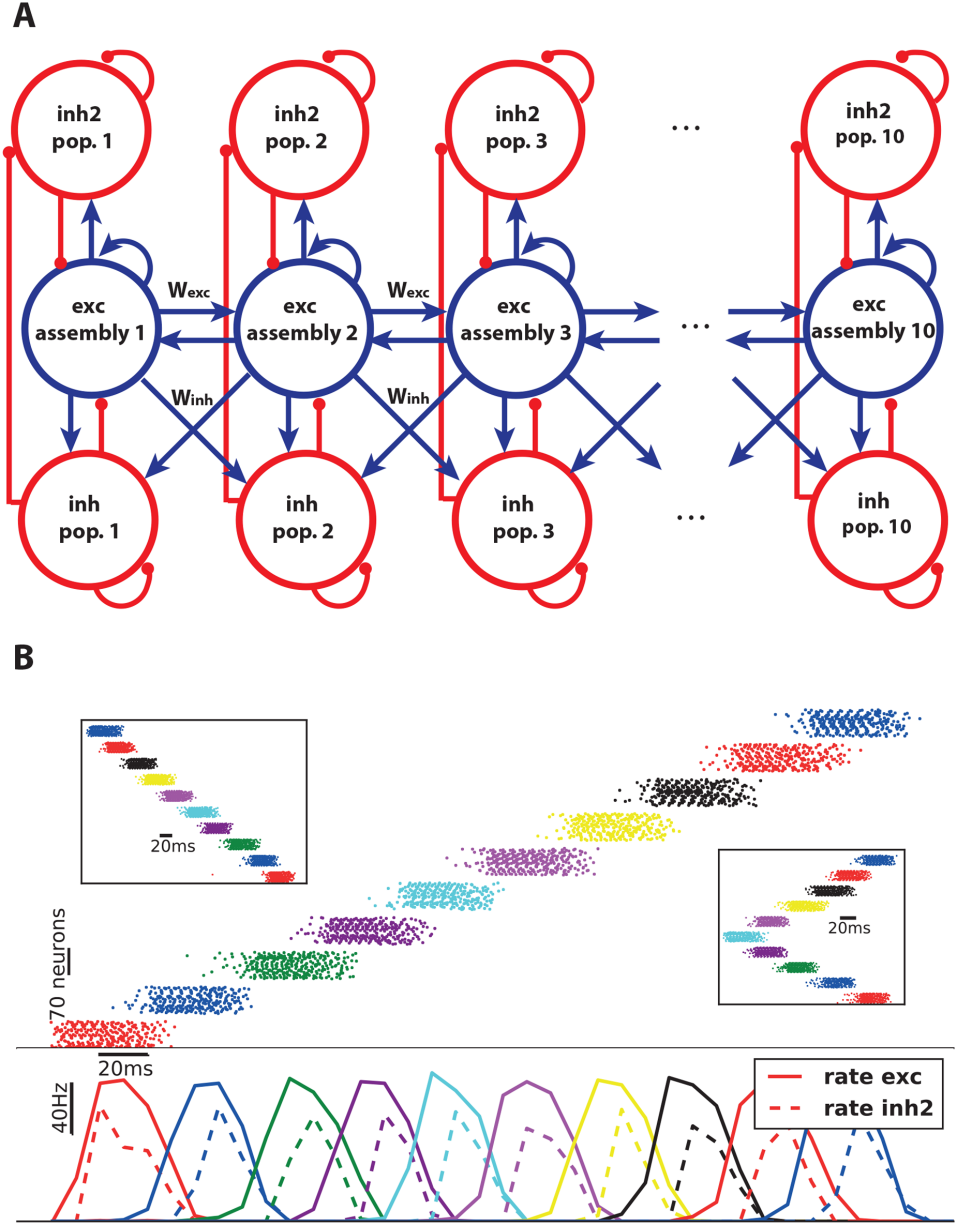
Reduction of firing rate by using two types of inhibitory neurons. **A**) A second type of inhibitory populations (Inh2) is added to control the high firing rate state. **B**) The firing rate of assembly neurons in the presence of new inhibitory neurons reduces to half the previous values (Fig 1B), while the chain is still able to propagate the activity forward, backward and in both directions.

The firing rate of assembly neurons during the active state is high (∼ 200Hz). In order to regulate the firing rate, we use a second type of inhibitory neurons for each assembly (Fig 4A). These additional inhibitory populations do not receive connections from the neighboring assemblies, but form only intra-group connections. Hence, they generate inhibition only after the assemblies become active, in contrast to the inhibitory groups in Fig 1. The idea of having the second type of inhibitory neurons is consistent with the observation that cortical networks contain different types of inhibitory neurons with different electrophysiological properties, different connectivity schema and probably different roles [40]. Fig 4B shows that in the presence of second inhibitory populations peak firing rates of excitatory neurons are reduced to ∼ 100Hz.

### A grid of assemblies as a skeleton for barrel cortex

**Fig 5:**
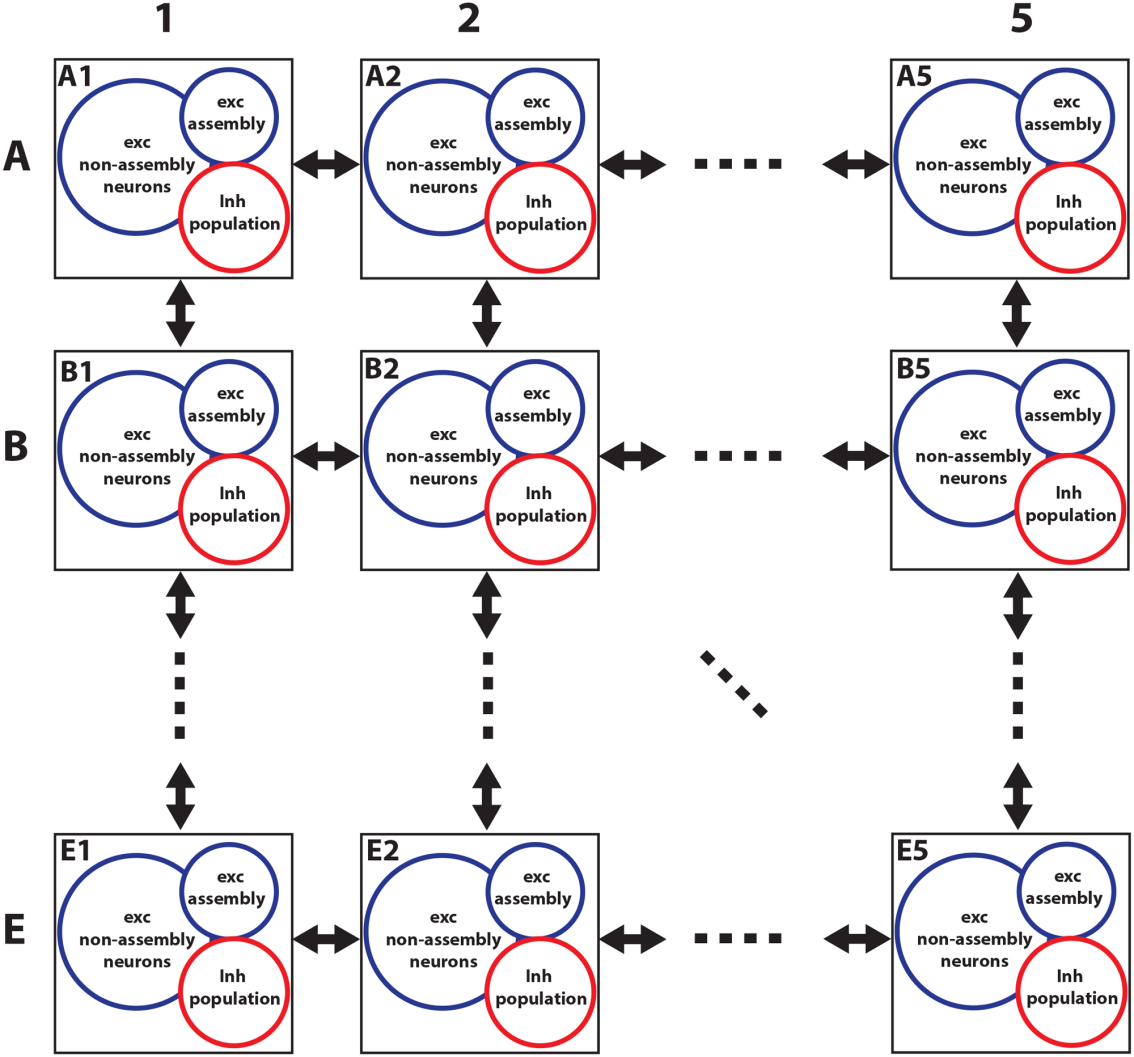
Schematic of a multi-column model of barrel cortex. Each column contains three groups: excitatory assembly and non-assembly groups, and an inhibitory group. Inside each column all groups are connected to each other, while between neighboring columns only excitatory neurons are connected. Non-neighboring columns have no connection in this model. One vertical line is called an ‘arc’, e.g. A2, B2, …, E2.

While the previous section focused on a one-dimensional structure, in this section we consider an excitation wave in a two-dimensional grid structure inspired by the layout of barrel cortex. Barrel cortex processes sensory information from the whiskers and is a part of the rodent somatosensory system. It consists of vertical modules called barrel columns, each of which relates to one principle whisker [41]. Here we make a multicolumn model of barrel cortex which contains 25 columns, organized in the shape of 5 arcs while each arc contains 5 rows. The actual mouse barrel cortex includes 33 columns with an arc of 4 rows, 4 arcs of 5 rows and 3 arcs of 3 rows [41]. For simplicity, we only consider one cortical layer of the barrel cortex. In our model, every column consists of excitatory and inhibitory neurons. Excitatory neurons are divided into two groups, a minority of assembly neurons and a majority of non-assembly neurons (see Table 2). Fig 5 shows the schematic of the model. While assembly neurons have high internal synaptic weights and connection probability, the connections between assembly and non-assembly neurons as well as connections inside non-assembly neurons are sparse and weak. Inside a column, all three groups have connections to each other, but inter-column connections are different from intra-column connections. (see Table 2). Columns are identical in terms of number of neurons, neural parameters and connections between neurons. Inhibitory neurons in our model form only intra-column connections and are not connected to the neurons of other columns, consistent with the common assumption that inhibitory neurons send short axons and contact only local targets [42]. In contrast, excitatory neurons of each column of our model connect to the excitatory neurons of the four nearest-neighbor columns. However, due to the relatively long distance between two neighboring columns, the connection probability between columns is taken as low (*p* = 10%), consistent with experimental observations [43].

Just as in the excitation chain of the previous section, the dynamics of each assembly can be explained by the three-fixed-point configuration. However, the subpopulations of non-assembly neurons have low network feedback coefficients (Eq 9) so that their dynamics exhibit only the low-activity fixed point. Hence the assemblies govern the dynamics of the grid model while non-assembly neurons follow the dynamics at a lower firing rate. Neglecting non-assembly and inhibitory neurons for a moment, we can consider this barrel cortex model merely as a grid of assembly neurons. We may think of this grid as the skeleton of our model for barrel cortex. Just as in the chain model, the synaptic weight between assemblies determines the propagation speed. Here, we adjusted the value of the inter-column synaptic weight (*w*_exc_ = 0.32mV) such that the speed of activity propagation in the model is similar to that observed in the experimental data [30, 31, 44]. We did not need to use short-term facilitation or depression to achieve the desired speed. The remaining parameters of the model are reported in section Materials and Methods.

#### Qualitative comparison with experiments

The grid model is able to reproduce several aspects of the dynamics of anesthetized barrel cortex in the stimulus-evoked and spontaneous regime. In the stimulus-evoked experiments [30, 45–47], a sensory signal was triggered by a brief deflection of a whisker. Voltage-sensitive dye imaging showed that the neural activity started in the barrel column corresponding to the stimulated whisker and propagated to the neighboring columns. After spreading over the whole field of view of barrel cortex, the activation vanished.

**Fig 6:**
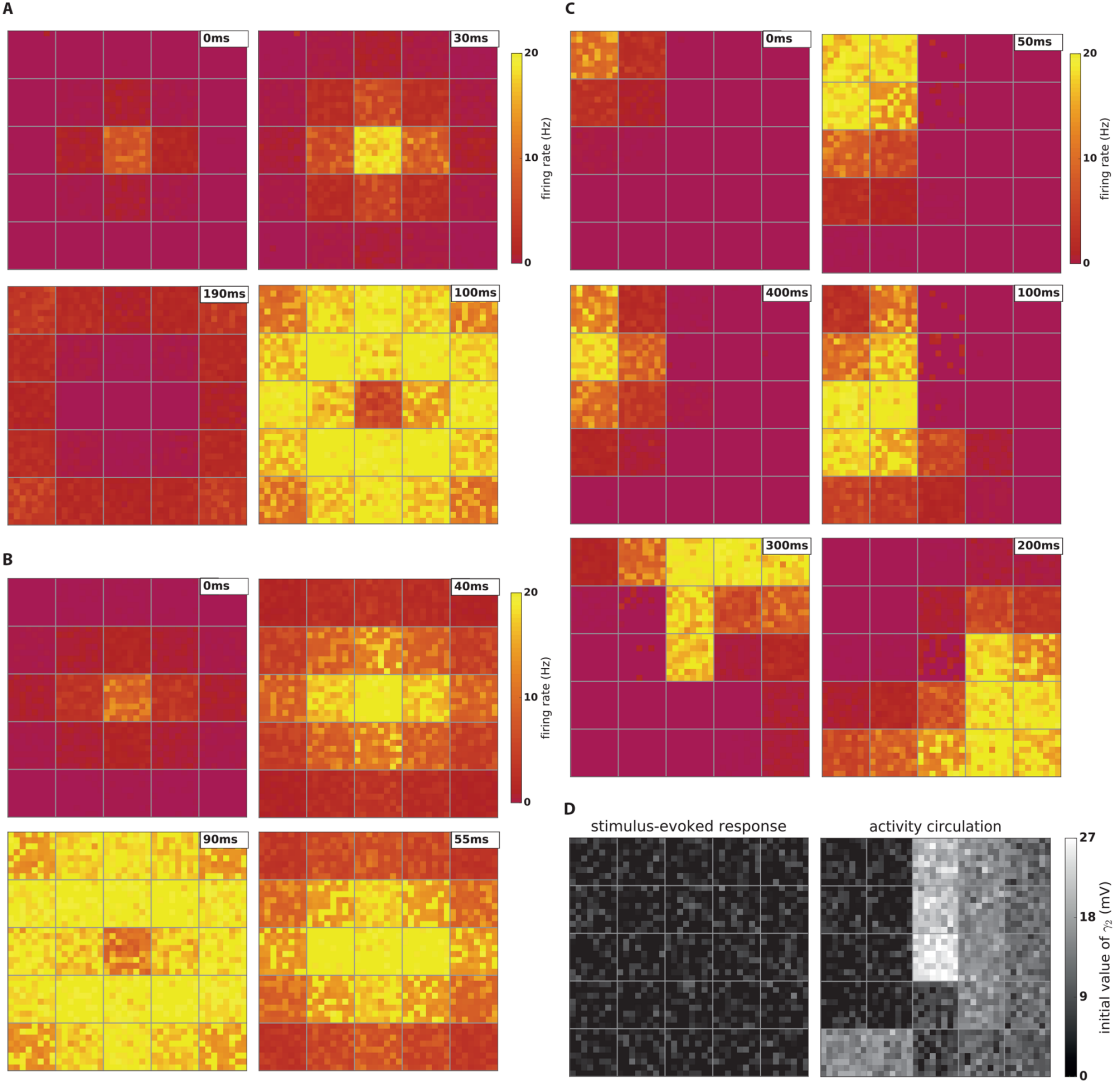
Multi-column model. **A**) Dynamics of the multi-column model after stimulation of the central column. The activity spreads over the barrel cortex. Each square in the figure shows 64 neurons randomly chosen from all three neuronal groups of a column. **B**) Similar to (**A**) with different connection probabilities between vertical (10%) and horizontal neighbors (15%). The difference in connectivity causes different propagation speed along the row compared to the speed along the arc. Note that in (**A**) both connectivities were 10% and the propagation speeds were identical. **C**) Circulation of activity in the multi-column model. After stimulation of a corner column, the activity propagates between columns and circulates across the model for several rounds. Eventually activity vanishes. This type of dynamic has been observed in spontaneous activity in mouse barrel cortex in vivo [31]. **D**) Initial values of the second adaptation kernel (*γ*_2_), which describes spike-triggered movement of the firing threshold (see Eq 3). For the stimulus-evoked response (shown in **A**) all initial values are drawn from a normal distribution with zero mean and standard deviation of 3mV. Any negative values are clamped to zero. For activity circulation (shown in **C**) we select the initial values to create a preferred direction of motion of the activity wave as follows. For each column, we first choose a mean value for the normal distribution, while the standard deviation of all distributions is the same (3mV). Negative values are clamped to zero. A high value of the mean adaptation variable for a column makes it dormant and does not allow it to activate until it recovers from the dormant mode. A low value, however, makes it excitable and ready to propagate the activity.

Fig 6A shows the simulated evolution of neuronal firing rate in the grid model after stimulating the neurons of the central column. The dynamics of the model are similar to the experimental recording. For better visibility, the voltage traces of the model were temporally filtered with a Gaussian function (*σ* = 30ms). We show only 64 neurons of each column: each panel in Fig 6A shows 25 squares (= columns) and each square contains 64 pixels (= neurons). These neurons are randomly selected from all neurons of the column (excitatory assembly and non-assembly neurons and inhibitory neurons). While the assembly neurons receive a high amount of synaptic input (due to their strong synaptic weights) and show a high firing rate, non-assembly and inhibitory neurons receive weaker weights and show lower firing rates (0–20Hz). Since assembly neurons form only a minority of all neurons in a column (see Materials and methods), the mean firing rate averaged across all neurons in a column is close to the firing rate of non-assembly and inhibitory neurons.

For the case of spontaneous activity, experiments showed that neural activity started on one side of the barrel cortex and only in a few columns. Then it moved from column to column in a circular fashion [31, 44]. With an appropriate initial state, our model is able to reproduce the circulation of activity, on the same time scale as in experiments. If we stimulate a model column on the upper-left side, the activity starts circulating to the down- and the leftward direction (Fig 6C). The activity orbits around the central column several times before it terminates.

Petersen et al. [30] found that in some experiments, after the stimulation, the activity spread faster along the row (horizontal spread in our model) than the arc (vertical spread in our model). The authors suggested that this could be caused by axons of excitatory neurons that extended farther along a row than along an arc. For an alternative interpretation of this phenomenon we assume that the connection probability between columns connected in the row direction (*p* = 15%) is greater than the connection probability along the arc direction (*p* = 10%). Simulations of this modified model exhibit a higher propagation speed along rows comparing to arcs (Fig 6B).

#### Qualitative analysis of activity wave

To understand the model dynamics we focus again on a regular grid of assemblies. As an aside we note that non-assembly neurons are subsidiary and passively follow assemblies. Since they receive weak synaptic input, they fire at a lower rate than neurons inside an assembly. We added non-assembly neurons in order to have the same number of neurons as observed in experiments [27] and to lower the mean firing rate averaged across a column to realistic values; but the dynamics of the network is solely driven by the assemblies. Similar to the assemblies of the excitation chain, each grid assembly is either in the dormant or the excitable mode depending on the adaptation level of its neurons. The dormant mode corresponds to high values of neuronal adaptation variables (spike-triggered current and firing threshold). Neurons of an assembly enter the dormant mode once they have emitted a burst of spikes (or if high initial values have been assigned to these variables). After recovering from the dormant mode, neurons of the assembly have a weak spike-triggered current and relatively low firing threshold. Hence the assembly has returned to the excitable mode and can now generate a burst of spikes in case of sufficient excitation. After activation, it switches again to the dormant mode.

In our model, we manipulate the initial value of the firing threshold kernel with longer time constant (*γ*_2_(*t*)) of neurons in the assembly in order to set the initial mode of each assembly. High initial values cause the dormant mode, while low initial values correspond to the excitable mode. Note that the kernel with shorter time constant (*γ*_1_(*t*)) is not suitable for this kind of manipulation, because the value of the kernel converges rapidly to zero.

For simulating the stimulus-evoked response we initialize all assemblies in the excitable mode. The initial value of *γ*_2_(*t*) of each neuron is randomly selected from a Gaussian distribution with zero-mean and *σ* = 3mV. All negative values are clipped to zero (Fig 6D (left)). After stimulation of the central assembly, it converges to the high point and produces a burst of spikes. As a result, neighboring assemblies receive some synaptic input. Although the value of this input current is low due to low inter-column connectivity, it suffices for the neighboring assemblies to pass the switch point and rapidly converge to the high point. This scenario repeats, and so the activation spreads over all assemblies. The assemblies transit to the dormant mode after a burst of activity, consistent with experimental data [30].

The simulation of activity circulation is more complicated and needs careful tuning of the initial values of the adaptation variables of the neurons. Figuratively speaking we carve a path for the activation by choosing suitable initial values for the firing thresholds (Fig 6D (right)). For neurons inside each column, we choose again initial values randomly from a Gaussian distribution with *σ* = 3mV. The distribution’s mean is different in each column. The means are selected such that there is path of excitable assemblies from the top-left of the grid to the column below the center. The activity propagates only along the path of excitable assemblies, whereas dormant assemblies do not switch to the high point even when they receive synaptic current. Once the activity has passed through the initially excitable assemblies, these become dormant. At the same time, the assemblies that were initially dormant recover so that the activity continues its path. This phenomenon repeats and causes activity circulating of the whole grid. After several rounds the circulation terminates because of a shortcut problem (see below). Non-assembly and inhibitory neurons do not contribute to shape the circular activation pattern. Instead, when they receive the activation from the assembly of their own column, they show some depolarization and a few spikes.

**Fig 7:**
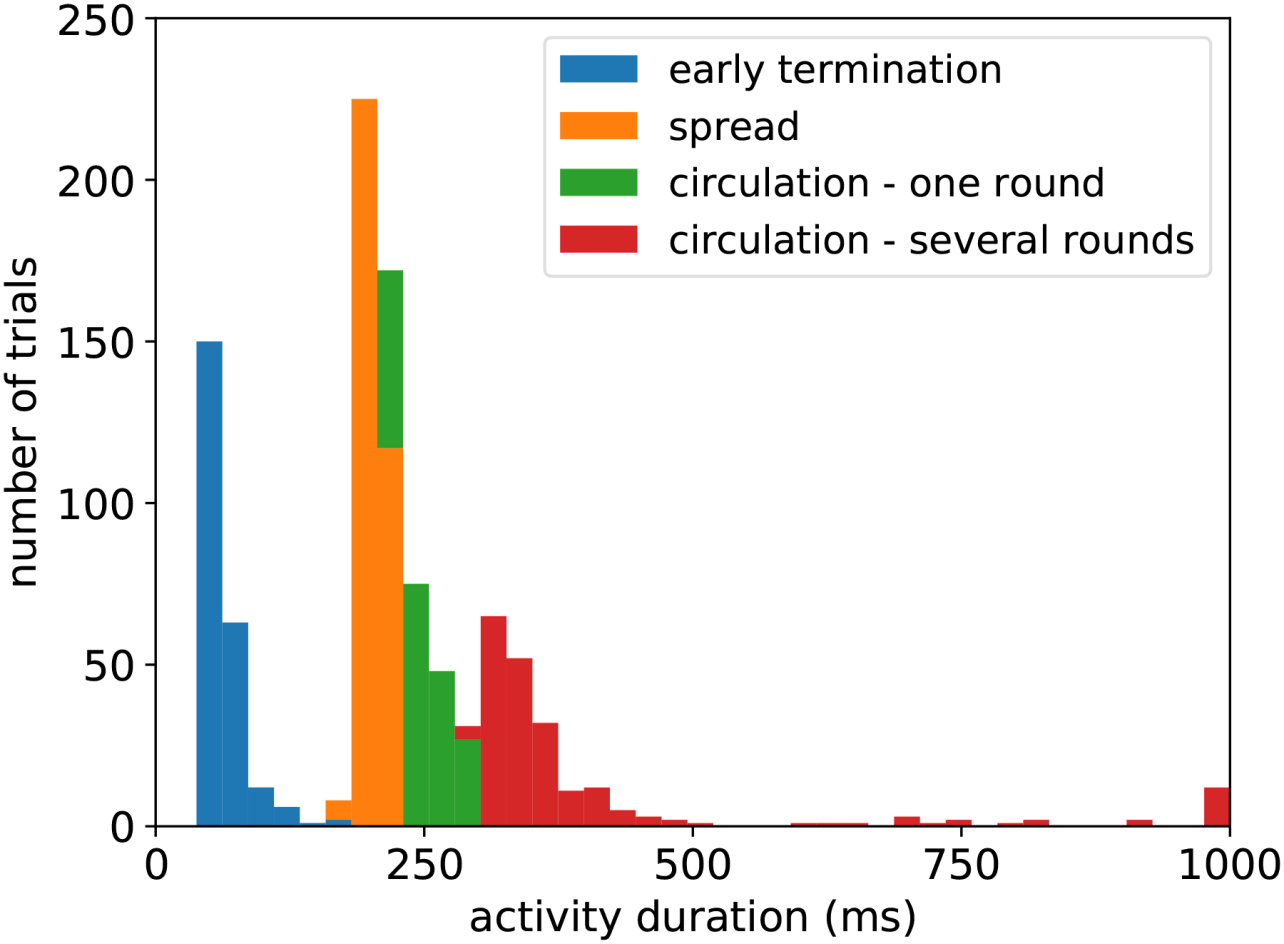
Effect of initial values on dynamics of the grid. Histogram of activity survival durations for 1000 trials of grid simulations with different initial values of the second adaptation kernel (*γ*_2_). For each trial we took the initial values shown in Fig 6D (right) and shuffled the columns. In ∼23% of trials the activity ceases before reaching all columns. In ∼35% of trials we observed that the activity spreads instead of circulating. In the remaining trials, we observed circulation with different patterns and durations depending on the path carved by the choice of initial values. The durations longer than 1000ms are clamped to 1000ms for better visibility.

One may think that pre-shaping an activation pattern by initialization is artificial. However, any distribution of initial values which make several neighboring assemblies excitable and leave others in the dormant mode has potentially such a “shaping” effect on the potential trajectory of the activity. Different patterns of initial values lead to different patterns of activity propagation observed in the cortex. In other words, we suggest that the great trial-to-trial variability of activity previously observed via voltage-sensitive dye imaging in the cortex [48, 49] might be due to different initial values of neurons in each trial. Sofar, we have tuned our pattern to construct a specific path; but in order to investigate the role of initial values more systematically, we have simulated 1000 trials. For the initial values of each trial, we randomly chose a shuffled version of initial values shown in Fig 6D (right). Specifically, we shuffled the mean of initial values of each column, but used a random distribution with *σ* = 3mV around the desired mean for neurons inside each column. Fig 7 indicates the types of activity propagation and the durations that activity survives in the grid. In ∼ 23% of trials the activity does not spread over all columns. In ∼ 35% of trials the activity spreads over all columns without repetition. In the rest of trials, cortical columns form a path of activation and the activity circulates for one or several rounds.

#### Termination of activity

Even with a highly tuned path, the activity terminates after several rounds. In order to understand the reason for this, we compare the activity path in early rounds and late rounds (S3 Fig). In the early rounds, the activity orbits around the central assembly and passes through all marginal assemblies to eventually reach the starting point at the corner again. Since, in our simulations, the central assembly has been assigned a very high initial value for the neuronal firing thresholds, it does not synchronize with its neighbors and does not initially follow them. However, after a while the central assembly becomes excitable and eventually activates when its, say, right-sided neighbor becomes active. Thereby it creates a shortcut for the activity which reaches the left-sided assemblies before the recovery from the dormant mode is complete. Since these assemblies are not recovered yet, they cannot become active. Therefore, the activity terminates and does not circulate any further. If we remove the central assembly or prevent it from being excitable (e.g. by decreasing its network feedback or increasing its intra-column inhibition), the activity can circulate for a very long time (green dots in S4 Fig). This is also the reason why we used absorbing boundaries for the grid. In case of reflecting boundaries (i.e., the activation wave reflects when it reaches the boundary) assemblies in the dormant mode do not have enough time for recovery and cause termination of the wave. We consider the alternative of periodic boundary conditions (i.e., when the activation wave passes a boundary, it jumps to the other side of grid) as not realistic, because once activity passes the boundary of a cortical area in the cortex, it generates (weak) propagation into a neighboring area rather than returning to the same area. Hence, we consider absorbing boundary as a good compromise and a step toward a very large scale model of neocortex, but simulating a large scale model is beyond this study.

As we described above, in our model the circulation of activity terminates spontaneously. This is consistent with spontaneous barrel cortex dynamics observed in vivo [31]. More specifically, every time the activity vanishes in the model, there is need for stimulation of a corner assembly which is in the excitable mode. If such stimulation is provided, the activity can circulate again for several rounds. Such a stimulus could potentially be provided by other cortical areas adjacent to the barrel cortex by reverberations of activity in thalamo-cortical loops or by novel whisker stimulation.

## Discussion

We have shown that a network of spiking neurons consisting of a chain of 11 assemblies can generate reliable temporally structured activity, that extends around one second, similar to excitable media on discrete structures [50]. While two well-known models of activity propagation, the synfire chain and rate propagation, require a feedforward network structure, our excitation chain works with a bidirectional activity pattern, similar to excitable media [22–24, 50]. This is advantageous because there is no direct experimental confirmation for the existence of systematic feedforward connectivity patterns in the brain [1–3, 7]. Moreover, the biophysical prerequisites of the excitation chain model, namely neuronal clustering, spike-frequency adaptation, recurrent connectivity and short-range inhibitory connections, are in principle consistent with experimental findings [29, 32, 38, 42]. Recent experimental evidence suggests a temporal evolution of activity over 500–1500ms [51, 52].

The excitation chain carries the signal by a wave of synchronous activity. Seen from this perspective, it is more similar to the synfire chain than to the fluctuating rate propagation which relies on asynchronous activity and rate coding [6]. However, the excitation chain is fundamentally different from the synfire chain and rate propagation in its ability to adjust the propagation speed without modifying the synaptic delay or synaptic time constant. While the propagation speed is nearly constant in a synfire chain [53], and is very close to the synaptic time scale in fluctuating rate propagation [7] and two-dimensional systems of neurons [54], we can slow down and adjust the speed in the excitation chain by changing the strength of inter-assembly synapses. Hence, we suggest that the excitation chain can be used for population coding across slow time scales [55, 56]. It is also possible that changes in synaptic weights are caused by neuro-modulators or various forms of activity-dependent synaptic plasticity. Suppose that a part of a neuronal structure which produces a complex cognitive behavior is an excitation chain, such that each assembly is responsible for performing one primitive of the behavior. Repeating the behavior, the assemblies become active one after each other in a systematic and reliable manner. In this case, a Hebbian learning rule will strengthen the synapses between excitatory assemblies and thus will work towards an increase of the execution speed of the behavior as we observed in Figs 1 and 2. This may explain why practicing sequential or rhythmic movements, such as in playing a musical instrument, can increase the speed at which the movement can be performed.

The bistability of neuronal assemblies is a key component of the excitation chain model. While the mechanism of assembly activation (jumping to the high point) is based on network feedback and is independent from the neuron model, the return of the assembly to the low point (in the dormant mode) needs an element of fatigue. In our model, we use spike-frequency adaptation for this purpose. However, replacing adaption by short-term depression [35] of intra-assembly synapses yields similar results. In a previous abstract [57], we removed spike-frequency adaptation and built a chain using leaky integrate-and-fire neurons while the synapses within each assembly expressed short-term depression. With strong connections inside assemblies, and relatively weak connections between them, the chain could propagate the activation both forward and backward. Similar to what is presented here, the propagation speed was tunable by changing the inter-assembly maximum synaptic weights. In a related model, a feedforward chain of neural assemblies with strong recurrent synapses which express short-term depression will propagate activity with propagation latency over 10 assemblies between 90ms and 200ms [9]. In contrast to this earlier model, we use symmetric connectivity so that propagation has no preferred direction. Another related work [58] studied the influence of short-term synaptic dynamics on switching activity patterns in networks that contain attractors.

One may wonder about the plausibility of neural assemblies in cortical networks. Applying synaptic plasticity rules in a randomly connected network in a balanced state [59, 60] leads to subgroups of neuron with strong recurrent connections [61]. A recent study used spike-timing dependent plasticity rules acting on excitatory to excitatory [62] and inhibitory to excitatory connections [63] as well as a synaptic scaling rule [64]. It was found that neurons which initially fire at higher firing rates compared to other neurons form subgroups with strong inward synapses and larger mean of outward synaptic weights after applying plasticity rules [61].

One way of extending our work is embedding the excitation chain in a bigger background network [5, 16, 17]. Then, excitatory assemblies and inhibitory populations receive synaptic input from the neurons of the larger background network. In case of a balanced background network, we would expect that the chain will exhibit results similar to those shown here, except that due to variance of synaptic input, the activation times will show more jitter compared to the result we obtained (Figs 1D, 1E and 2C). Constructing a balanced network for adapting neurons will be possible using recent mean field methods [65] and is left for future work. It would also be interesting to further explore bidirectional synaptic connections between excitatory and additional inhibitory neurons within the same group. Our expectation is that, if parameters are tuned to get a balanced state, the peak firing rate of neurons inside the assembly would be significantly reduced without a substantial change in the speed of signal propagation across the chain; cf. Fig 4.

In the grid model, assemblies are responsible for transmitting the activity between barrel columns. Removing the inhibitory and non-assembly neurons from the model, the grid of assemblies is able to propagate and circulate the activity on its own. Therefore we consider the grid of assemblies as the skeleton of the model, while the other neurons just follow the activity wave. This, however, is not meant to imply that inhibitory and non-assembly neurons do not play an important role in the cortex. Whereas in our model, we only consider the anesthetized state, information processing in the awake cortex very likely involves more than just the assembly neurons and requires the contribution of the other neurons.

In the literature, there exist several competition-based models for reproducing cortical trial-to-trial variability [66–69] and for modeling working memory using continuous attractors [70, 71]. In these models, individual neurons or neural assemblies (also called neural clusters) try to become active and to suppress others by direct inhibitory connections or global inhibition. However, in the two models considered here (the chain and grid of assemblies) the situation is completely different. Instead of competition, the assemblies cooperate with each other to propagate activity signals through the tissue. Hence, in this type of model there is no need for global inhibition.

Similar to bump attractor models proposed for implementing working memory [70, 71] and attractor maps used for encoding spatial location in hippocampus [72–74], our model relies on strong recurrent excitatory connections. However, while these encoding-models are designed to stabilize neuronal activity in time in order to provide a stationary memory of a continuous variable, our model’s aim is to propagate the activity with a specified speed and path to relay sensory information or to perform slow behavioral tasks.

Activity propagation was studied previously using the Wilson-Cowan model of neuronal population dynamics [22, 23] and the dynamics of neural fields [24, 25, 50, 75, 76]. However, it is often hard to link network parameters used in these abstract models to neuronal and synaptic parameters. In our approach, we start directly from neural parameters extracted from experiments [38, 39] and explore the influence of synaptic weight or short-term plasticity [35]. Despite the richness of the biological parameter space, the essential mathematical features of excitable media [22–25] are still apparent in the models examined here. In the literature, there exist several studies [77, 78] that observed traveling activity waves in a balanced network with distance dependent connectivity structure.

The multicolumn model presented in this work is able to produce dynamics similar to the spontaneous regime of cortical activity [31]. However, it is different from resting-state networks [79–83] designed for explaining the synchrony of different brain regions. In these models, each brain region is typically considered as a nonlinear oscillator. The coupling strength between oscillators [79] as well as synaptic transmission delay and noise [80, 81] are tuned such that different brain regions show synchrony similar to observed data. While such models are very successful in representing resting-state activity, their focus on macroscopic network structure may limit the range of dynamics produced by the models. In contrast, in our model we focus on a particular element of neuronal circuit-level connectivity, namely local assemblies of neurons, which may be contained in a small part of each cortical column. While such assemblies may make only a small contribution to the average activity, macroscopic propagation of activity across barrel columns or brain regions may depend on such assemblies. In systems with excitable elements such as ours, the type of dynamics strongly depends on initial conditions of the neurons and nonlinearly on the inputs [84]. Therefore embedded excitable assemblies provide nolinear mesoscopic processing characteristics to the circuits that may easily be overlooked in resting-state or other macroscopic models relying on averaged measures of connectivity between areas.

To conclude we would like to highlight the predictive aspect of our work. So far, neural assemblies have been used for explaining working memory [70, 71], cortical trial-to-trial variability [66–69] and slow oscillations in the cortex [34]. We suggest that beside these roles, neural assemblies are also responsible for activity with adjustable speed in the cortex [52]. Consider a complex behavioral task which includes a sequence of several subtasks. If activation of each assembly of the chain initiates a subtask, the whole chain is able to perform the complex tasks with desirable speed. In case of repeating the tasks, Hebbian learning strengthens interassembly synapses and faster propagation. Therefore, the cortical network is able to perform the task faster after practicing the task. Because of symmetric connectivity, we could explain bidirectional activity propagation observed in the experiments [32]. Furthermore, our work predicts that inhibitory neurons reduce the propagation speed. In a multi-array recording [32] with GABA receptor antagonists near the electrodes of array, we therefore predict an increase in the propagation speed compared to the control condition.

## Materials and methods

### Neuron model and population parameters

Neuronal parameters used in the simulations are reported in Table 1. Table 2 summarizes the network parameters.

**Table 1:** Neuron model parameters used in simulations.

Parameter	Excitatory	Inhibitory	Inhibitory2*
*C* (pF)	63.0	54.2	29.4
*g*_*L*_ (nS)	8.1	5.5	2.9
*E*_*L*_ (mV)	−58.6	−59.8	-61.6
*η*(*t*)	*η*_1_(*t*) + *η*_2_(*t*)	*η*_1_(*t*) + *η*_2_(*t*)	*η*_1_(*t*) + *η*_2_(*t*)
*η*_1_(*t*) (pA) for (*t* ≥ 0)	36.3*e*^−*t*/39.2ms^	47.4*e*^−*t*/19.1ms^	15.2*e*^−*t*/13.2ms^
*η*_2_(*t*) (pA) for (*t* ≥ 0)	0.7*e*^−*t*/700.0ms^	−0.8*e*^−*t*/282.1ms^	16.2*e*^−*t*/70.7ms^
*γ*(*t*)	*γ*_1_(*t*) + *γ*_2_(*t*)	*γ*_1_(*t*) + *γ*_2_(*t*)	*γ*_1_(*t*) + *γ*_2_(*t*)
*γ*_1_(*t*) (mV) for (*t* ≥ 0)	3.26*e*^−*t*/45.0ms^ (**)	−7.3*e*^−*t*/28.3ms^	5.8*e*^−*t*/31.9ms^
*γ*_2_(*t*) (mV) for (*t* ≥ 0)	2.52*e*^−*t*/204.3ms^	3.7*e*^−*t*/347.7ms^	1.9*e*^−*t*/382.2ms^
λ_0_ (Hz)	0.1	0.1	0.1
Δ*V* (mV)	1.76	1.24	1.30
$V_{T}^{*}$ (mV)	−56	−44.5	-56.1
*τ*_ref_ (ms)	4.0	4.0	4.0
*V*_reset_ (mV)	−31.9	−38.7	-40.2

(*) Only applicable for Fig 4.

(**) This value is 13.05*e*^−*t*/45.0ms^ for the simulation of the multicolumn model (Fig 6).

**Table 2:** Parameters of networks used in simulations (exc: excitatory, inh: inhibitory, pop: population, amp: amplitude, CP: connection probability, PSP: postsynaptic potential, PSC: postsynaptic current, assem: assembly, conn: connections).

Simulation of excitation chain (Figs 1 and 2)					
Size of exc. assem.	70				
Size of inh. pop.*	70				
	Conn.	PSP amp.(mV)	PSC amp.(pA)	CP	*τ*_syn_(ms)
Inter-column conn.	exc. to exc.	Variable	Variable	10%	7.7
	exc. to inh.*	Variable	Variable	30%	9.9
Intra-column conn.	exc. to exc.	1.0	22.1	50%	7.7
	exc. to inh.*	0.25	3.7	10%	9.9
	inh. to exc.*	0.16	3.5	22%	7.7
	inh. to inh.*	0.56	10.2	30%	6.7
	exc. to inh2.**	0.49	7.3	30%	9.9
	inh2. to exc.**	0.22	5.0	50%	7.7
	inh2. to inh2.**	0.56	10.2	30%	6.7
	inh. to inh2.**	1.12	20.4	30%	6.7
* Not applicable for the chain of only excitatory assemblies (Fig 2)					
** Only applicable for the chain containing two types of inhibitory neurons (Fig 4)					
Simulation of multicolumn barrel cortex model (Fig 6)					
Size of exc. assem.	70				
Size of exc. non-assem.	380				
Size of inh. pop.	70				
	Conn.	PSP amp.(mV)	PSC amp.(pA)	CP	*τ*_syn_(ms)
Inter-column conn.	exc. to exc.*	0.32	7.1	10%	7.7
Inter-column conn.	assem. to assem.	1.0	22.1	50%	7.7
	assem. to non-assem.	0.10	2.2	10%	7.7
	non-assem. to non-assem.	0.15	3.3	15%	7.7
	non-assem. to assem.	0.10	2.2	10%	7.7
	inh. to exc.*	0.16	3.5	22%	7.7
	exc. to inh.*	0.25	3.7	10%	9.9
	inh. to inh.	0.56	10.2	30%	6.7
* exc. indicates both assembly and non-assembly groups.					

As our neuron model we use a current-based generalized integrate-and-fire (GIF) model which implements spike-frequency adaptation using a spike-triggered current and a moving firing-threshold mechanisms [38]. The dynamics of the neuron’s sub-threshold membrane potential (*V*(*t*)) is described by:

$$\begin{array}{c} C\frac{dV(t)}{dt}=-g_{L}\left(V\left(t\right)-E_{L}\right)-\sum_{\hat{t_{j}}<t}\eta \left(t-\hat{t_{j}}\right)+I\left(t\right) \end{array}$$ (1)

where parameters *C*, *g*_*L*_ and *E*_*L*_ are the passive parameters of the neuron. *I*(*t*) is the synaptic input and *η*(*t*) is the shape of the spike-triggered current caused by spikes of the neuron itself at times ${\hat{t}}_{j}$. After each spike emission, the membrane potential is reset to *V*_reset_, integration of Eq 1 restarts and the neuron goes through an absolute refractory period of duration *τ*_ref_.

Spikes are produced stochastically (similar to an inhomogeneous Poisson process) with the firing intensity:

$$\begin{array}{c} \lambda \left(t\right)=\lambda_{0}\text{exp}(\frac{V\left(t\right)-V_{\text{T}}\left(t\right)}{\Delta V}) \end{array}$$ (2)

where λ_0_ is the stochastic intensity at the firing threshold *V*_T_, and Δ*V* is a constant which defines the level of stochasticity. The threshold *V*_T_ follows the dynamic:

$$\begin{array}{c} V_{\text{T}}\left(t\right)=V_{\text{T}}^{*}+\sum_{\hat{t_{j}}\;<\;t}\gamma \left(t-\hat{t_{j}}\right) \end{array}$$ (3)

where $V_{\text{T}}^{*}$ is constant and *γ*(*t*) describes the time course of the threshold after a spike emission. In Eq (1), the synaptic input *I*_*i*_(*t*) received by neuron *i* is determined by the spikes of synaptically connected neurons:

$$\begin{array}{c} I_{i}\left(t\right)=\sum_{j}w_{ij}\sum_{f}\alpha \left(t-t_{j}^{f}\right)=\sum_{j}w_{ij}\int_{0}^{\infty }\alpha \left(s\right)S_{j}\left(t-s\right)ds \end{array}$$ (4)

where *w*_*ij*_ is the weight of the synapse connecting neuron *j* to neuron *i*, and $\alpha \left(t\right)=e^{-\left(t-\Delta \right)/\tau_{\text{syn}}}$ for *t* ≥ Δ is the post-synaptic current (PSC) shape. The synaptic transmission delay (Δ) in all simulation is 1ms. $S_{j}\left(t\right)=\sum_{k}\delta \left(t-t_{j}^{k}\right)$ is the spike train of neuron *j*, *δ* denotes the Dirac *δ*-function and $t_{j}^{k}$ is the *k*^th^ spike of neuron *j*. The synaptic weight *w*_*ij*_ indicates the PSC amplitude. Given the neuronal parameters, one can relate the PSC to the post-synaptic potential (PSP). We report values both of PSC and PSP amplitudes used in our simulations.

We ran simulations using the Brian simulator [85] for simulating the chain and NEST [86] for simulating the grid.

### Transient stimulus

In order to initiate the activity in the chain, we stimulate one assembly of the chain (for example the first assembly, the last assembly or an assembly in the middle of the chain). For stimulating an assembly, we connect each of its neurons to 25 Poisson neurons which fire with the rate of 5Hz each for 25ms. The synaptic weight between the Poisson neurons and the assembly neurons is 0.18nA.

### Rate-current relations

Here we present some theory necessary to explain the dynamics of excitable assemblies. The dynamics of a neuronal population can be described by two equations relating the firing rate averaged over all neurons and the mean of the synaptic input received by them. The first relation is called the neuron’s gain function. Injecting a weakly fluctuating current *I*_syn_ into a neuron produces an average firing rate of

$$\begin{array}{c} r=g(\langle I_{\text{syn}}\rangle ,\sigma_{\text{I}}) \end{array}$$ (5)

where 〈*I*_syn_〉 and *σ*_I_ are the average and the standard deviation of the synaptic current over time, respectively, and *g* is the gain function. Although there are ways to compute the firing rate of adaptive integrate-and-fire neuron models in closed-form [87, 88] or by using a self-consistent numerical approach [89–91], there is no straightforward analytical solution for computing the gain function of the GIF model that we use here. We obtain the gain function (5) by numerical simulation [34]. For the simulations to determine the gain function numerically the injected current is given by

$$\begin{array}{c} I\left(t\right)=\langle I_{\text{syn}}\rangle +\frac{\sigma_{\text{I}}}{\sqrt{q_{2}}}\int_{0}^{\infty }\alpha \left(s\right)\xi \left(t-s\right)ds \end{array}$$ (6)

where *ξ*(*t*) is white noise with mean 〈*ξ*(*t*)〉 = 0 and covariance 〈*ξ*(*t*)*ξ*(*t*′)〉 = *δ*(*t* − *t*′), *α*(*t*) is the shape of the PSC defined above and $q_{2}=\int_{0}^{\infty }\alpha^{2}\left(t\right)dt$. Depending on the duration of injection, the neuron goes into different adaptation states. By injecting the current (6) for a short episode of 10ms, we can estimate the firing rate in the non-adapted state. In case of a longer stimulation period, we can divide the time into intervals of 10ms and extract the rate-current relation in the different, progressively more adapted states. This method has been used to obtain the gain functions displayed in Fig 3A and 3B.

The network activity gives rise to the second relation between the average firing rate and the average synaptic current. The synaptic input of an arbitrary neuron *i* is described by:

$$\begin{array}{c} I_{i,\text{syn}=}\sum_{j}w_{ij}(\int_{0}^{\infty }\alpha \left(s\right)S_{j}\left(t-s\right)ds) \end{array}$$ (7)

where *w*_*ij*_ is the weight of the synapse connecting neuron *j* to neuron *i* and *S*_*j*_(*t*) is the spike train of neuron *j*. The sum runs over all other neurons *j* in the assembly. Averaging both sides over time and input neurons gives the average input current: 〈*I*_syn_〉 = *Npqwr*, where *N* is the number of neurons inside the population, *p* is the connection probability between neurons, *w* is the synaptic weight and *q* is the total charge of one PSC pulse: $q=\int_{0}^{\infty }\alpha \left(t\right)dt$. Rearranging this equation yields:

$$\begin{array}{c} r=\frac{\langle I_{\text{syn}}\rangle }{Npqw} \end{array}$$ (8)

We refer to the denominator of Eq 8 as the network feedback coefficient (*C*_fb_) of the population [34]:

$$\begin{array}{c} C_{\text{fb}}=Npqw \end{array}$$ (9)

We use these two relations for analysis of the behavior of excitatory assemblies in the “Results” section.

### Analytical approach for obtaining the propagation speed of the excitation chain

Our aim is to estimate the difference between activation times of two consecutive assemblies (which is a measure of propagation speed) using an analytical approach. We assume that the values of the parameters are given and the time course *r*(*t*) of the population rate signal of an arbitrary assembly in the chain is known. Furthermore, we assume that the populations are silent before activation (*r*(*t*) = 0 for *t* < *t*_a_).

Suppose that we have two excitatory assemblies, assembly 1 and assembly 2. We refer to their activation time as ${\bar{t}}_{1}$ and ${\bar{t}}_{2}$ respectively. The aim of the calculation is to find the difference in activation times $x={\bar{t}}_{2}-{\bar{t}}_{1}$. Recall that the activation time has been defined above as the expected time at which all assembly neurons have spiked once. Assuming independent Poisson firing of the assembly with rate *r*(*t*), we can find ${\bar{t}}_{1}$ as the moment when the expected spike count reaches 1, $n(0;\;{\bar{t}}_{1})/N=1$, where *n*(*a*; *b*) is the number of spikes in time interval [*a*, *b*) and *N* is the number of neuron in the assembly. Inserting the shape of assembly population rate we obtain

$$\begin{array}{ccc} \int_{0}^{{\bar{t}}_{1}}r\left(t\right)dt & = & 1 \end{array}$$ (10)

which we can solve for ${\bar{t}}_{1}$. Note, however, that the Poisson assumption made here does not ensure that no neuron fires more than one spike before ${\bar{t}}_{1}$. Therefore Eq 10 yields an approximate value of ${\bar{t}}_{1}$.

Finding the value of ${\bar{t}}_{2}$ is more complicated. After activation of assembly 1, its neurons send synaptic input to assembly2. The average input received by each assembly2 neuron can be computed:

$$\begin{array}{c} I_{\text{syn\_fwd}}\left(t\right)=Np_{\text{exc}}w_{\text{exc}}\left(\alpha \left(t\right)*r\left(t-\Delta \right)\right) \end{array}$$ (11)

where *p*_exc_ and *w*_exc_ are inter-assemblies connection probability and synaptic weight respectively, * denotes the convolution operator and Δ is synaptic transmission delay. However, this is not the only synaptic input received by neurons in assembly2. Even before ${\bar{t}}_{2}$, several neurons of assembly2 may already fire spikes (due to random fluctuations) and send some feedback current to other neurons. This averaged current can be computed similarly:

$$\begin{array}{c} I_{\text{syn\_self}}\left(t\right)=\left(N-1\right)p_{\text{self}}w_{\text{self}}\left(\alpha \left(t\right)*r\left(t-\Delta -x\right)\right) \end{array}$$ (12)

where *p*_self_ and *w*_self_ are intra-assemblies connection probability and synaptic weight respectively, and *x* is the difference of activation times. Note that the shape of *r*(*t*) is assumed to be the same for all assemblies of the chain. However, we must be careful about the timing of each current. Suppose that at the time *t* = 0 assembly 1 starts to fire, therefore assembly2 receives a first input at the time *t* = Δ. After a while, assembly2 starts to fire. The difference between these two starting times is denoted by *x*. Therefore the feedback current is received by assembly2 neurons at the time *t* = Δ + *x*.

The total synaptic input received by neurons of assembly2 is the summation of *I*_syn_fwd_ and *I*_syn_self_. Consequently, we can write the total input received by assembly2 neurons as

$$\begin{array}{c} I_{\text{syn}}\left(t\right)=I_{\text{syn\_fwd}}\left(t\right)+I_{\text{syn\_self}}\left(t\right) \end{array}$$ (13)

Now we can calculate the subthreshold membrane potential of neurons in assembly2 by solving Eq 1:

$$\begin{array}{c} V\left(t\right)=E_{\text{L}}+\frac{1}{C}\left(e^{-t/\tau_{\text{m}}}*I_{\text{syn}}\left(t\right)\right) \end{array}$$ (14)

Note that since we want to calculate the time of first spikes of neurons, we can neglect the spike-triggered current and the moving firing threshold. The firing intensity is then given as a function of *V*(*t*) by Eq (2), except that $V_{\text{T}}\left(t\right)=V_{\text{T}}^{*}$ because we assumed that neurons are not adapted and all *γ*(*t*) equal zero.

Then using the distribution of first spikes *P*(*t*), we are able to calculate the average time of first spikes of assembly2:

$$\begin{array}{c} P\left(t\right)=\lambda \left(t\right)\text{exp}(-\int_{0}^{t}\lambda \left(t^{\prime }\right)dt^{\prime }) \end{array}$$ (15)

$$\begin{array}{c} {\bar{t}}_{2}=\int_{0}^{\infty }tP\left(t\right)dt \end{array}$$ (16)

Finally, using Eqs 10 and 16, we can calculate the time difference between activation times:

$$\begin{array}{c} x={\bar{t}}_{2}-{\bar{t}}_{1} \end{array}$$ (17)

Note, however, that ${\bar{t}}_{2}$ in Eq (17) depends on the value of *x* through Eq (12). Therefore, we have formed a self-consistent equation for *x*. We feed this value in Eq 12 and get it back in Eq 17. If the output value of *x* equals its input value, we found the proper value. Using a simple search, we are able to find this value numerically.

We apply this approach for our chain and calculate the value of *x* for different values of *w*_exc_; these results are presented in Fig 3D. Note that we need to obtain the shape of the assembly population rate *r*(*t*) by neural simulation beforehand. However, the same shape *r*(*t*) can be used for all values of *w*_exc_, because for *p*_exc_*w*_exc_ ≪ *p*_self_*w*_self_ the time course of the initial rise is dominated by the self-feedback (see Fig 3B).

### Short-term plasticity

We use short-term plasticity [35, 36] in one series of our simulations (Fig 1F). This synaptic model supposes that each synapse has a certain amount of resources (such as neurotransmitter packed in vesicles ready for release) denoted by *x*, with dynamics

$$\begin{array}{c} \frac{dx}{dt}=\frac{1-x}{\tau_{\text{rec}}}-u\;x\;\delta \left(t-t_{f}\right) \end{array}$$ (18)

$$\begin{array}{c} \frac{du}{dt}=\frac{U-u}{\tau_{\text{facil}}}+U\left(1-u\right)\delta \left(t-t_{f}\right) \end{array}$$ (19)

where *U* (jump of release fraction), *τ*_rec_ (recovery time constant) and *τ*_facil_ (facilitation time constant) are three parameters of the model. *u* (release fraction) and *x* are the two variables of the system. Whenever a neuron fires a spike (*t*_*f*_ denotes the firing time), it produces a PSC with amplitude of *uxw* (*w* is the synaptic weight). Then, the amount of resource is decreased by *ux* and the release fraction is increased by *U*(1 − *u*). In our simulations, we either used facilitation or depression. We chose the values *τ*_rec_ = 0.001ms and *τ*_facil_ = 500ms for the facilitation case and *τ*_rec_ = 800ms and *τ*_facil_ = 0.001ms for the depression case. The value of *U* is different in each simulation. Note that for the depression case, we have to fix the amplitude of the first PSC regardless of value of *U*. We did that by adjusting the value of the synaptic weight.

### Shuffling initial values on grid

We simulate different trials of the grid with shuffled initial values (Fig 7). The aim of shuffling is to show that many initial patterns will lead to activity circulation and not just the tuned pattern of Fig 6D (right). For the shuffling, we take mean values of *γ*_2_(0) (initial value of the moving threshold kernel with longer time constant) of each column in Fig 6D (right) and randomly assign these means to columns. Then, using these means, we randomly choose the value of *γ*_2_(0) for each neuron in the columns.

## Supporting information

S1 Fig Different transient stimuli. The propagation speed is indepenent of transient stimuli properties. Modification of the number of Poisson neurons (**A**) and the synaptic weight between Poisson neurons and the first assembly (**B**) does not affect the propagation speed. The chain and its parameters are the same as Fig 6A.
(TIFF)

S2 Fig Different time constants of short term plasticity. Changing recovery time constant of short term depression (*τ*_rec_, red points) and time constant of short term facilitation (*τ*_facil_, blue points) does not affect the propagation speed. The chain and its parameters are the same as Fig 6A.
(TIFF)

S3 Fig Termination of activity circulation in the grid. The activity circulation in the grid terminates after several rounds because of a short circuit in central assemblies (C4→C3→C2). In early rounds the difference of activation time between C4 and C3 are shorter compared to the late rounds (red ellipses). In other words, C3 becomes active sooner than it is expected. Therefore, C3 is able to activate C2 (red arrow), while C2 is supposed to be activated by B2. This short circuit generates activity before assemblies recover from the dormant mode. Hence, other assemblies are not able to become active and the circulation ceases. The initial values are the same as shown in Fig 6D (right). Column A1 is stimulated at *t* = 100ms in order to start the circulation.
(TIFF)

S4 Fig Longer activity circulation in the grid. Long duration of circulation by removing column C3. We repeat the simulation of S3 Fig with same condition except that we removed column C3. Green dots show the dynamics of the new configuration while we keep blue dots from the S3 Fig for better comparison. In the new configuration, the circulation runs for much longer. In the figure, we have only shown the first two seconds, but we have not seen termination for 10 seconds.
(TIFF)

## References

[1] Abeles M. Role of the cortical neuron: integrator or coincidence detector? Isr J Med Sci. 1982;18(1):83–92. 6279540

[2] Abeles M. Corticonics: Neural circuits of the cerebral cortex. Cambridge University Press; 1991.

[3] Diesmann M, Gewaltig MO, Aertsen A. Stable propagation of synchronous spiking in cortical neural networks. Nature. 1999;402(6761):529–533. 10.1038/990101 10591212

[4] Gewaltig MO. Evolution of synchronous spike volleys in cortical Networks: Network simulations and continuous probabilistic models. Shaker; 2000.

[5] Kumar A, Rotter S, Aertsen A. Conditions for propagating synchronous spiking and asynchronous firing rates in a cortical network model. J Neurosci. 2008;28(20):5268–5280. 10.1523/JNEUROSCI.2542-07.2008 18480283 PMC6670637

[6] Kumar A, Rotter S, Aertsen A. Spiking activity propagation in neuronal networks: reconciling different perspectives on neural coding. Nat Rev Neurosci. 2010;11(9):615–627. 10.1038/nrn2886 20725095

[7] van Rossum MC, Turrigiano GG, Nelson SB. Fast propagation of firing rates through layered networks of noisy neurons. J Neurosci. 2002;22(5):1956–1966. 10.1523/JNEUROSCI.22-05-01956.2002 11880526 PMC6758872

[8] Vogels T, Abbott L. Signal propagation and logic gating in networks of integrate-and-fire neurons. J Neurosci. 2005;25(46):10786–10795. 10.1523/JNEUROSCI.3508-05.2005 16291952 PMC6725859

[9] van Rossum MC, van der Meer MA, Xiao D, W M. Adaptive integration in the visual cortex by depressing recurrent cortical circuits. Neural Comput. 2008;20(7):1847–1872. 10.1162/neco.2008.06-07-546 18336081

[10] Kistler WM, Gerstner W. Stable propagation of activity pulses in populations of spiking neurons. Neural Comput. 2002;14(5):987–997. 10.1162/089976602753633358 11972904

[11] Goedeke S, Diesmann M. The mechanism of synchronization in feed-forward neuronal networks. New J Phys. 2008;10(1):015007 10.1088/1367-2630/10/1/015007

[12] Schrader S, Grün S, Diesmann M, Gerstein GL. Detecting synfire chain activity using massively parallel spike train recording. J Neurophysiol. 2008;100(4):2165–2176. 10.1152/jn.01245.2007 18632888 PMC2576207

[13] Torre E, Canova C, Denker M, Gerstein G, Helias M, Grün S. ASSET: analysis of sequences of synchronous events in massively parallel spike trains. PLoS Comput Biol. 2016;12(7):e1004939 10.1371/journal.pcbi.1004939 27420734 PMC4946788

[14] Hahnloser RH, Kozhevnikov AA, Fee MS. An ultra-sparse code underliesthe generation of neural sequences in a songbird. Nature. 2002;419(6902):65–70. 10.1038/nature00974 12214232

[15] Oram M, Wiener M, Lestienne R, Richmond B. Stochastic nature of precisely timed spike patterns in visual system neuronal responses. J Neurophysiol. 1999;81(6):3021–3033. 10.1152/jn.1999.81.6.3021 10368417

[16] Hanuschkin A, Diesmann M, Morrison A. A reafferent and feed-forward model of song syntax generation in the Bengalese finch. J Comput Neurosci. 2011;31(3):509–532. 10.1007/s10827-011-0318-z 21404048 PMC3232349

[17] Hanuschkin A, Herrmann JM, Morrison A, Diesmann M. Compositionality of arm movements can be realized by propagating synchrony. J Comput Neurosci. 2011;30(3):675–697. 10.1007/s10827-010-0285-9 20953686 PMC3108016

[18] Trengove C, van Leeuwen C, Diesmann M. High-capacity embedding of synfire chains in a cortical network model. J Comput Neurosci. 2013;34(2):185–209. 10.1007/s10827-012-0413-9 22878688 PMC3605496

[19] Trengove C, Diesmann M, Leeuwen CV. Dynamic effective connectivity in cortically embedded systems of recurrently coupled synfire chains. J Comput Neurosci. 2016;40(1):1–26. 10.1007/s10827-015-0581-5 26560334 PMC4762935

[20] Gerstner W, Ritz R, Hemmen JLV. A biologically motivated and analytically soluble model of collective oscillations in the cortex. Biol Cybern. 1993;68(4):363–374. 10.1007/BF00201861 8386552

[21] Izhikevich EM. Polychronization: computation with spikes. Neural Comput. 2006;18(2):245–282. 10.1162/089976606775093882 16378515

[22] Wilson HR, Cowan JD. Excitatory and inhibitory interactions in localized populations of model neurons. Biophys J. 1972;12(1):1–24. 10.1016/S0006-3495(72)86068-5 4332108 PMC1484078

[23] Wilson HR, Cowan JD. A mathematical theory of the functional dynamics of cortical and thalamic nervous tissue. Biol Cybern. 1973;13(2):55–80. 10.1007/BF00288786 4767470

[24] Jirsa VK, Haken H. A derivation of a macroscopic field theory of the brain from the quasi-microscopic neural dynamics. Physica D: Nonlinear Phenomena. 1997;99(4):503–526. 10.1016/S0167-2789(96)00166-2

[25] Coombes S. Neural fields. Scholarpedia. 2006;1(6):1373 10.4249/scholarpedia.1373

[26] Capone C, Rebollo B, Muñoz A, Illa X, Giudice PD, Sanchez-Vives MV, et al Slow Waves in Cortical Slices: How Spontaneous Activity is Shaped by Laminar Structure. Cereb Cortex. 2017; p. 1–17. 10.1093/cercor/bhx326 29190336

[27] Lefort S, Tomm C, Sarria JCF, Petersen CCH. The Excitatory Neuronal Network of the C2 Barrel Column in Mouse Primary Somatosensory Cortex. Neuron. 2009;61(2):301–316. 10.1016/j.neuron.2008.12.020 19186171

[28] Avermann M, Tomm C, Mateo C, Gerstner W, Petersen CCH. Microcircuits of excitatory and inhibitory neurons in layer 2/3 of mouse barrel cortex. J Neurophysiol. 2012;107(11):3116–3134. 10.1152/jn.00917.2011 22402650

[29] Perin R, Berger TK, Markram H. A synaptic organizing principle for cortical neuronal groups. Proc Natl Acad Sci USA. 2011;108(13):5419–5424. 10.1073/pnas.1016051108 21383177 PMC3069183

[30] Petersen CC, Grinvald A, Sakmann B. Spatiotemporal dynamics of sensory responses in layer 2/3 of rat barrel cortex measured in vivo by voltage-sensitive dye imaging combined with whole-cell voltage recordings and neuron reconstructions. J Neurosci. 2003;23(4):1298–1309. 10.1523/JNEUROSCI.23-04-01298.2003 12598618 PMC6742278

[31] Petersen CC, Hahn TT, Mehta M, Grinvald A, Sakmann B. Interaction of sensory responses with spontaneous depolarization in layer 2/3 barrel cortex. Proc Natl Acad Sci USA. 2003;100(23):13638–13643. 10.1073/pnas.2235811100 14595013 PMC263866

[32] Sanchez-Vives MV, McCormick DA. Cellular and network mechanisms of rhythmic recurrent activity in neocortex. Nat Neurosci. 2000;3(10):1027–1034. 10.1038/79848 11017176

[33] Giugliano M, Camera GL, Fusi S, Senn W. The response of cortical neurons to in vivo-like input current: theory and experiment: II. Time-varying and spatially distributed inputs. Biol Cybern. 2008;99(4-5):303–318. 10.1007/s00422-008-0270-9 19011920

[34] Setareh H, Deger M, Petersen CCH, Gerstner W. Cortical dynamics in presence of assemblies of densely connected weight-hub neurons. Front Comput Neurosci. 2017;11:52 10.3389/fncom.2017.00052 28690508 PMC5480278

[35] Tsodyks MV, Markram H. The neural code between neocortical pyramidal neurons depends on neurotransmitter release probability. Proc Natl Acad Sci USA. 1997;94(2):719–723. 10.1073/pnas.94.2.719 9012851 PMC19580

[36] Tsodyks M, Pawelzik K, Markram H. Neural Networks with Dynamic Synapses. Neural Comput. 1998;10(4):821–835. 10.1162/089976698300017502 9573407

[37] Pozzorini C, Naud R, Mensi S, Gerstner W. Temporal whitening by power-law adaptation in neocortical neurons. Nat Neurosci. 2013;16(7):942–948. 10.1038/nn.3431 23749146

[38] Mensi S, Naud R, Pozzorini C, Avermann M, Petersen CC, Gerstner W. Parameter extraction and classification of three cortical neuron types reveals two distinct adaptation mechanisms. J Neurophysiol. 2012;107(6):1756–1775. 10.1152/jn.00408.2011 22157113

[39] Pozzorini C, Mensi S, Hagens O, Naud R, Koch C, Gerstner W. Automated high-throughput characterization of single neurons by means of simplified spiking models. PLoS Comput Biol. 2015;11(6):e1004275 10.1371/journal.pcbi.1004275 26083597 PMC4470831

[40] Rudy B, Fishell G, Lee S, Hjerling-Leffler J. Three groups of interneurons account for nearly 100% of neocortical GABAergic neurons. Dev Neurobiol. 2011;71(1):45–61. 10.1002/dneu.20853 21154909 PMC3556905

[41] Petersen CC. The functional organization of the barrel cortex. Neuron. 2007;56(2):339–355. 10.1016/j.neuron.2007.09.017 17964250

[42] Fino E, Packer AM, Yuste R. The logic of inhibitory connectivity in the neocortex. The Neuroscientist. 2013;19(3):228–237. 10.1177/1073858412456743 22922685 PMC4133777

[43] Boucsein C, Nawrot M, Schnepel P, Aertsen A. Beyond the cortical column: abundance and physiology of horizontal connections imply a strong role for inputs from the surround. Front Neurosci. 2011;5:32 10.3389/fnins.2011.00032 21503145 PMC3072165

[44] Ferezou I, Haiss F, Gentet LJ, Aronoff R, Weber B, CH C. Spatiotemporal dynamics of cortical sensorimotor integration in behaving mice. Neuron. 2007;56(5):907–923. 10.1016/j.neuron.2007.10.007 18054865

[45] Civillico EF, Contreras D. Integration of evoked responses in supragranular cortex studied with optical recordings in vivo. J Neurophysiol. 2006;96(1):336–351. 10.1152/jn.00128.2006 16571736

[46] Ferezou I, Bolea S, Petersen CC. Visualizing the cortical representation of whisker touch: voltage-sensitive dye imaging in freely moving mice. Neuron. 2006;50(4):617–629. 10.1016/j.neuron.2006.03.043 16701211

[47] Lustig BR, Friedman RM, Winberry JE, Ebner FF, Roe AW. Voltage-sensitive dye imaging reveals shifting spatiotemporal spread of whisker-induced activity in rat barrel cortex. J Neurophysiol. 2013;109(9):2382–2392. 10.1152/jn.00430.2012 23390314 PMC3652220

[48] Arieli A, Sterkin A, Grinvald A, Aertsen A. Dynamics of ongoing activity: explanation of the large variability in evoked cortical responses. Science. 1996;273(5283):1868 10.1126/science.273.5283.1868 8791593

[49] Kenet T, Bibitchkov D, Tsodyks M, Grinvald A, Arieli A. Spontaneously emerging cortical representations of visual attributes. Nature. 2003;425(6961):954–956. 10.1038/nature02078 14586468

[50] Murray JD. Mathematical Biology. II Spatial Models and Biomedical Applications {Interdisciplinary Applied Mathematics V. 18}. Springer-Verlag New York Incorporated; 2001.

[51] Schmitt LI, Wimmer RD, Nakajima M, Happ M, Mofakham S, Halassa MM. Thalamic amplification of cortical connectivity sustains attentional control. Nature. 2017;545(7653):219 10.1038/nature22073 28467827 PMC5570520

[52] Wang J, Narain D, Hosseini EA, Jazayeri M. Flexible timing by temporal scaling of cortical responses. Nat Neurosci. 2018;21(1):102 10.1038/s41593-017-0028-6 29203897 PMC5742028

[53] Wennekers T, Palm G. Controlling the speed of synfire chains. Artificial Neural Networks—ICANN. 1996; p. 451–456. 10.1007/3-540-61510-5_78

[54] Kistler WM, Seitz R, Hemmen JLV. Modeling collective excitations in cortical tissue. Physica D: Nonlinear Phenomena. 1998;114(3-4):273–295. 10.1016/S0167-2789(97)00195-4

[55] Buonomano DV, Maass W. State-dependent computations: spatiotemporal processing in cortical networks. Nat Rev Neurosci. 2009;10(2):113 10.1038/nrn2558 19145235

[56] Runyan CA, Piasini E, Panzeri S, Harvey CD. Distinct timescales of population coding across cortex. Nature. 2017;548(7665):92–96. 10.1038/nature23020 28723889 PMC5859334

[57] Setareh H, Deger M, Gerstner W. Synaptic efficacy tunes speed of activity propagation through chains of bistable neural assemblies. In: COSYNE 2015. EPFL-POSTER-206999; 2015.

[58] Bibitchkov D, Herrmann JM, Geisel T. Pattern storage and processing in attractor networks with short-time synaptic dynamics. Netw Comput Neural Syst. 2002;13(1):115–129. 10.1080/net.13.1.115.129 11873841

[59] Vreeswijk CV, Sompolinsky H. Chaos in neuronal networks with balanced excitatory and inhibitory activity. Science. 1996;274(5293):1724–1726. 10.1126/science.274.5293.1724 8939866

[60] Brunel N. Dynamics of sparsely connected networks of excitatory and inhibitory spiking neurons. J Comput Neurosci. 2000;8(3):183–208. 10.1023/A:1008925309027 10809012

[61] Effenberger F, Jost J, Levina A. Self-organization in balanced state networks by STDP and homeostatic plasticity. PLoS Comput Biol. 2015;11(9):e1004420 10.1371/journal.pcbi.1004420 26335425 PMC4559467

[62] Song S, Miller KD, Abbott LF. Competitive Hebbian learning through spike-timing-dependent synaptic plasticity. Nat Neurosci. 2000;3(9):919–926. 10.1038/78829 10966623

[63] Haas JS, Nowotny T, Abarbanel HD. Spike-timing-dependent plasticity of inhibitory synapses in the entorhinal cortex. J Neurophysiol. 2006;96(6):3305–3313. 10.1152/jn.00551.2006 16928795

[64] Pozo K, Goda Y. Unraveling mechanisms of homeostatic synaptic plasticity. Neuron. 2010;66(3):337–351. 10.1016/j.neuron.2010.04.028 20471348 PMC3021747

[65] Schwalger T, Deger M, Gerstner W. Towards a theory of cortical columns: From spiking neurons to interacting neural populations of finite size. PLoS Comput Biol. 2017;13(4):e1005507 10.1371/journal.pcbi.1005507 28422957 PMC5415267

[66] Litwin-Kumar A, Doiron B. Slow dynamics and high variability in balanced cortical networks with clustered connections. Nat Neurosci. 2012;15(11):1498–1505. 10.1038/nn.3220 23001062 PMC4106684

[67] Doiron B, Litwin-Kumar A. Balanced neural architecture and the idling brain. Fron Comput neurosci. 2014;8:56. 10.3389/fncom.2014.00056 PMC4034496 24904394

[68] Mazzucato L, Fontanini A, La Camera G. Dynamics of multistable states during ongoing and evoked cortical activity. J Neurosci. 2015;35(21):8214–8231. 10.1523/JNEUROSCI.4819-14.2015 26019337 PMC4444543

[69] Mazzucato L, Fontanini A, La Camera G. Stimuli reduce the dimensionality of cortical activity. Front Syst Neurosci. 2016 10.3389/fnsys.2016.00011 26924968 PMC4756130

[70] Compte A, Brunel N, Goldman-Rakic PS, Wang XJ. Synaptic mechanisms and network dynamics underlying spatial working memory in a cortical network model. Cereb Cortex. 2000;10(9):910–923. 10.1093/cercor/10.9.910 10982751

[71] Wang M, Yang Y, Wang CJ, Gamo NJ, Jin LE, Mazer JA, et al NMDA receptors subserve persistent neuronal firing during working memory in dorsolateral prefrontal cortex. Neuron. 2013;77(4):736–749. 10.1016/j.neuron.2012.12.032 23439125 PMC3584418

[72] Samsonovich A, McNaughton BL. Path integration and cognitive mapping in a continuous attractor neural network model. J Neurosci. 1997;17(15):5900–5920. 10.1523/JNEUROSCI.17-15-05900.1997 9221787 PMC6573219

[73] Tsodyks M. Attractor neural network models of spatial maps in hippocampus. Hippocampus. 1999;9(4):481–489. 10.1002/(SICI)1098-1063(1999)9:4<481::AID-HIPO14>3.0.CO;2-S 10495029

[74] McNaughton BL, Battaglia FP, Jensen O, Moser EI, Moser MB. Path integration and the neural basis of the ’cognitive map’. Nat Rev Neurosci. 2006;7(8):663–678. 10.1038/nrn1932 16858394

[75] Bressloff P. Synaptically generated wave propagation in excitable neural media. Phys Rev Lett. 1999;82(14):2979 10.1103/PhysRevLett.82.2979

[76] Coombes S. Waves, bumps, and patterns in neural field theories. Biol Cybern. 2005;93(2):91–108. 10.1007/s00422-005-0574-y 16059785

[77] Rosenbaum R, Doiron B. Balanced networks of spiking neurons with spatially dependent recurrent connections. Phys Rev X. 2014;4(2):021039.

[78] Pyle R, Rosenbaum R. Spatiotemporal dynamics and reliable computations in recurrent spiking neural networks. Phys Rev Lett. 2017;118(1):018103 10.1103/PhysRevLett.118.018103 28106418

[79] Honey CJ, Kötter R, Breakspear M, Sporns O. Network structure of cerebral cortex shapes functional connectivity on multiple time scales. Proc Natl Acad Sci USA. 2007;104(24):10240–10245. 10.1073/pnas.0701519104 17548818 PMC1891224

[80] Ghosh A, Rho Y, McIntosh AR, Kötter R, Jirsa VK. Noise during rest enables the exploration of the brain’s dynamic repertoire. PLoS Comput Biol. 2008;4(10):e1000196 10.1371/journal.pcbi.1000196 18846206 PMC2551736

[81] Deco G, Jirsa V, McIntosh AR, Sporns O, Kötter R. Key role of coupling, delay, and noise in resting brain fluctuations. Proc Natl Acad Sci USA. 2009;106(25):10302–10307. 10.1073/pnas.0901831106 19497858 PMC2690605

[82] Deco G, Jirsa VK, McIntosh AR. Emerging concepts for the dynamical organization of resting-state activity in the brain. Nat Rev Neurosci. 2011;12(1):43–56. 10.1038/nrn2961 21170073

[83] Gilson M, Moreno-Bote R, Ponce-Alvarez A, Ritter P, Deco G. Estimation of directed effective connectivity from fMRI functional connectivity hints at asymmetries of cortical connectome. PLoS Comput Biol. 2016;12(3):e1004762 10.1371/journal.pcbi.1004762 26982185 PMC4794215

[84] Helias M, Deger M, Rotter S, Diesmann M. Instantaneous non-linear processing by pulse-coupled threshold units. PLoS Comput Biol. 2010;6(9):e1000929 10.1371/journal.pcbi.1000929 20856583 PMC2936519

[85] Goodman D, Brette R. Brian: A Simulator for Spiking Neural Networks in Python. Front Neuroinform. 2008;2 10.3389/neuro.11.005.2008 19115011 PMC2605403

[86] Gewaltig MO, Diesmann M. NEST (neural simulation tool). Scholarpedia. 2007;2(4):1430 10.4249/scholarpedia.1430

[87] Fourcaud-Trocmé N, Hansel D, Vreeswijk CV, Brunel N. How spike generation mechanisms determine the neuronal response to fluctuating inputs. J Neurosci. 2003;23(37):11628–11640. 10.1523/JNEUROSCI.23-37-11628.2003 14684865 PMC6740955

[88] Hertäg L, Durstewitz D, Brunel N. Analytical approximations of the firing rate of an adaptive exponential integrate-and-fire neuron in the presence of synaptic noise. Front Comput Neurosci. 2014;8 10.3389/fncom.2014.00116 25278872 PMC4167001

[89] La Camera G, Rauch A, Lüscher HR, Senn W, Fusi S. Minimal models of adapted neuronal response to in Vivo-Like input currents. Neural Comput. 2004;16(10):2101–2124. 10.1162/0899766041732468 15333209

[90] Richardson MJ. Firing-rate response of linear and nonlinear integrate-and-fire neurons to modulated current-based and conductance-based synaptic drive. Phys Rev E. 2007;76(2):021919 10.1103/PhysRevE.76.021919 17930077

[91] Richardson MJ. Dynamics of populations and networks of neurons with voltage-activated and calcium-activated currents. Phys Rev E. 2009;80(2):021928 10.1103/PhysRevE.80.021928 19792172

