# Supplementary figures and images for: Excitable neuronal assemblies with adaptation as a building block of brain circuits for velocity-controlled signal propagation
Source: PLoS Comput Biol. 2018 Jul 6;14(7):e1006216. doi: 10.1371/journal.pcbi.1006216 (PMC6051644; doi:10.1371/journal.pcbi.1006216)

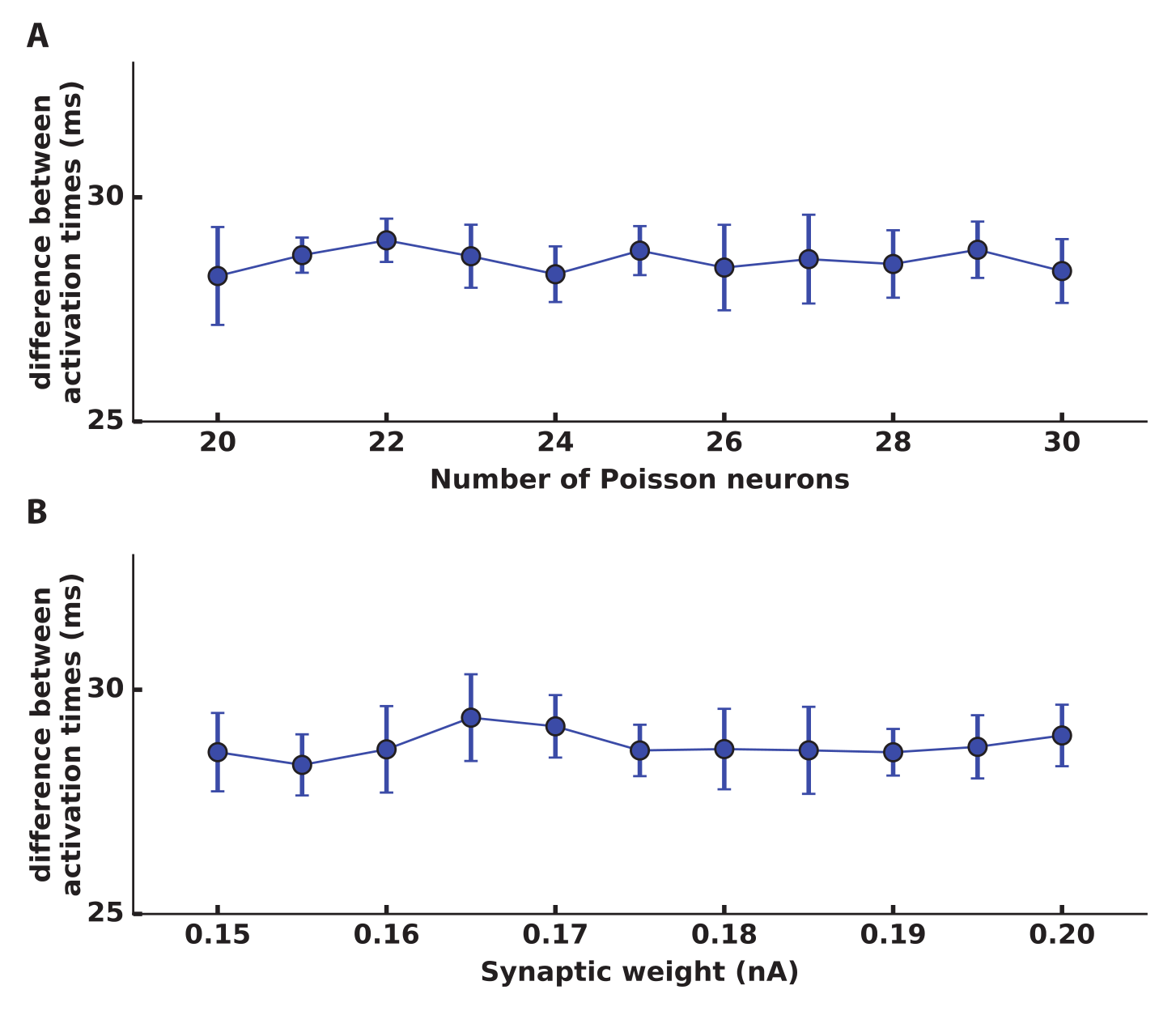

Supplement: S1 Fig — The propagation speed is indepenent of transient stimuli properties. Modification of the number of Poisson neurons (A) and the synaptic weight between Poisson neurons and the first assembly (B) does not affect the propagation speed. The chain and its parameters are the same as Fig 6A. (TIFF) [file pcbi.1006216.s001.tiff]

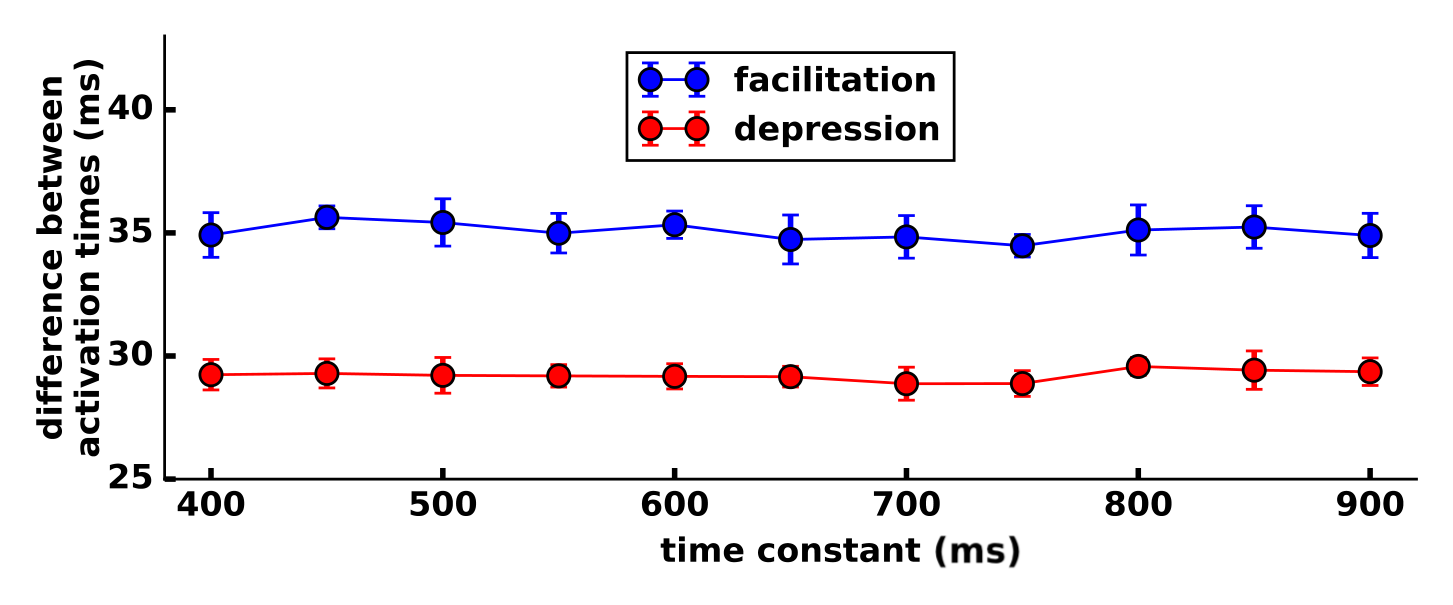

Supplement: S2 Fig — Changing recovery time constant of short term depression (τrec, red points) and time constant of short term facilitation (τfacil, blue points) does not affect the propagation speed. The chain and its parameters are the same as Fig 6A. (TIFF) [file pcbi.1006216.s002.tiff]

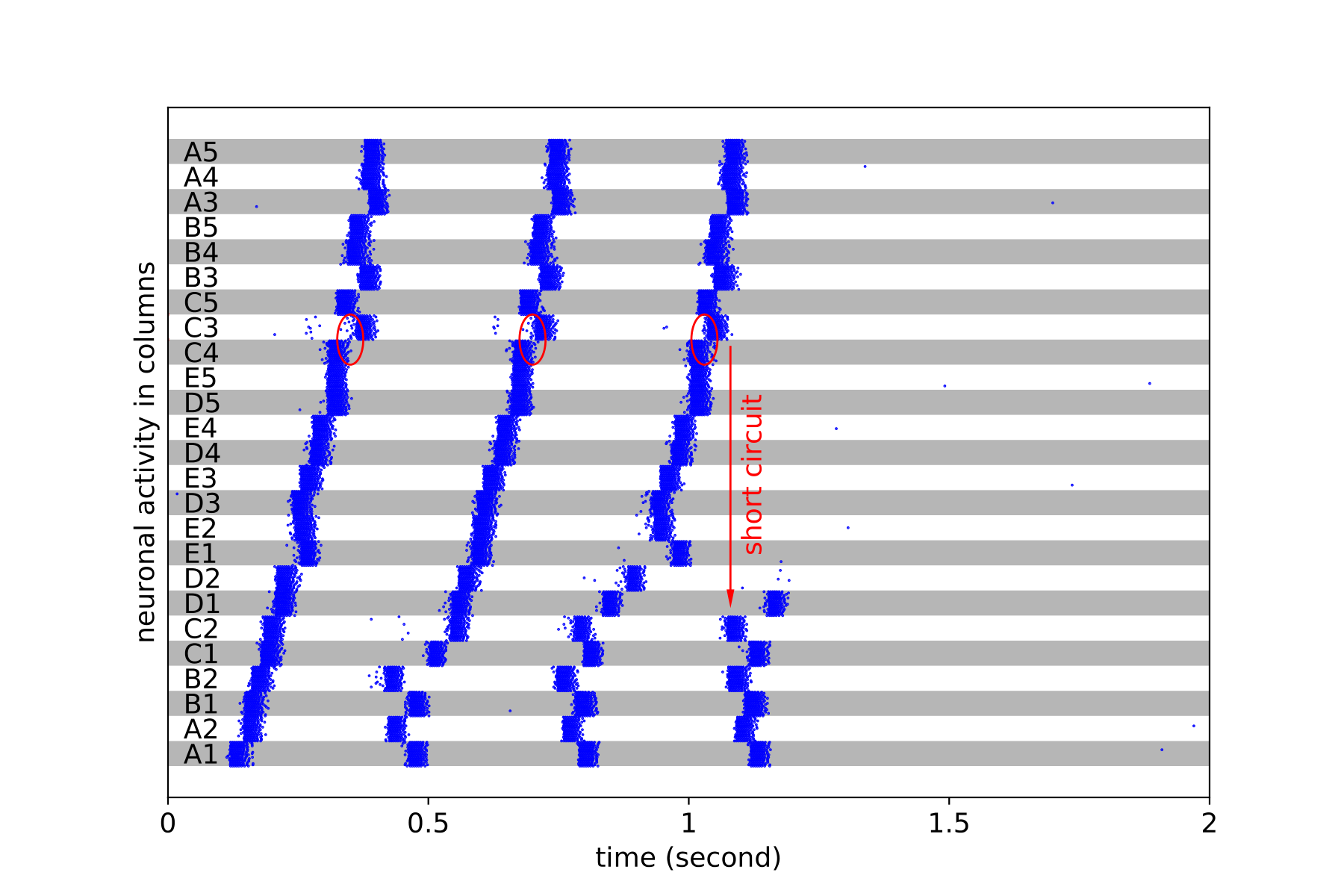

Supplement: S3 Fig — The activity circulation in the grid terminates after several rounds because of a short circuit in central assemblies (C4→C3→C2). In early rounds the difference of activation time between C4 and C3 are shorter compared to the late rounds (red ellipses). In other words, C3 becomes active sooner than it is expected. Therefore, C3 is able to activate C2 (red arrow), while C2 is supposed to be activated by B2. This short circuit generates activity before assemblies recover from the dormant mode. Hence, other assemblies are not able to become active and the circulation ceases. The initial values are the same as shown in Fig 6D (right). Column A1 is stimulated at t = 100ms in order to start the circulation. (TIFF) [file pcbi.1006216.s003.tiff]

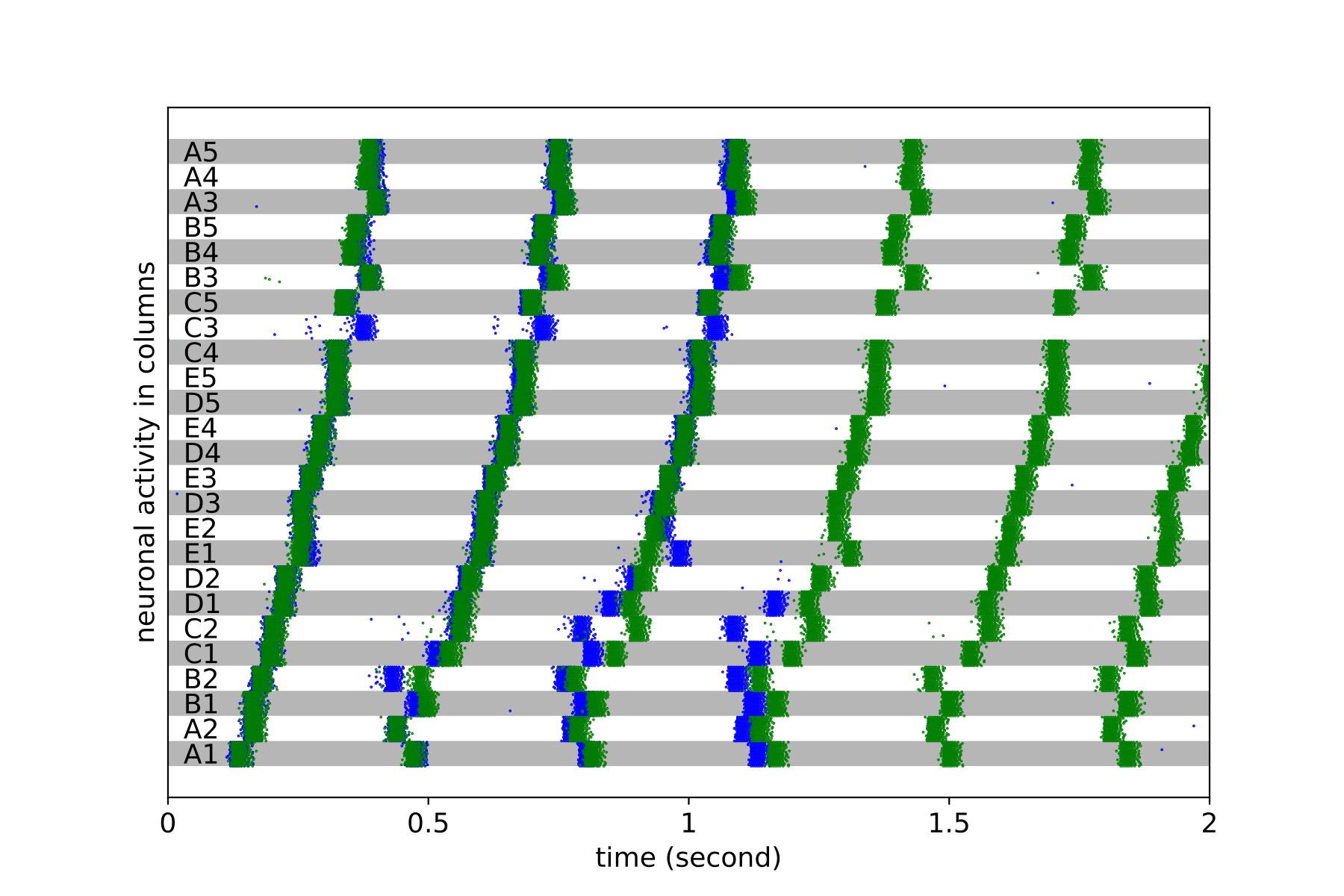

Supplement: S4 Fig — Long duration of circulation by removing column C3. We repeat the simulation of S3 Fig with same condition except that we removed column C3. Green dots show the dynamics of the new configuration while we keep blue dots from the S3 Fig for better comparison. In the new configuration, the circulation runs for much longer. In the figure, we have only shown the first two seconds, but we have not seen termination for 10 seconds. (TIFF) [file pcbi.1006216.s004.tiff]
